# Advances in nanomaterial-based targeted drug delivery systems

**DOI:** 10.3389/fbioe.2023.1177151

**Published:** 2023-04-13

**Authors:** Xiaoxiao Cheng, Qirong Xie, Yang Sun

**Affiliations:** Department of Ultrasound, The Second Affiliated Hospital of Chongqing Medical University, Chongqing Key Laboratory of Ultrasound Molecular Imaging, Institute of Ultrasound Imaging, Chongqing, China

**Keywords:** nanomaterials, nano-drug delivery system, surface modification, targeted delivery, EPR, blood brain barrier

## Abstract

Nanomaterial-based drug delivery systems (NBDDS) are widely used to improve the safety and therapeutic efficacy of encapsulated drugs due to their unique physicochemical and biological properties. By combining therapeutic drugs with nanoparticles using rational targeting pathways, nano-targeted delivery systems were created to overcome the main drawbacks of conventional drug treatment, including insufficient stability and solubility, lack of transmembrane transport, short circulation time, and undesirable toxic effects. Herein, we reviewed the recent developments in different targeting design strategies and therapeutic approaches employing various nanomaterial-based systems. We also discussed the challenges and perspectives of smart systems in precisely targeting different intravascular and extravascular diseases.

## 1 Introduction

In the past few years, the successful development of nanotechnology, especially the emergence of new nanomaterials, has provided new ideas and potential methods for diagnosing and treating many major diseases (Kinnear et al., 2017; Miguel et al., 2022). Because of their unique physicochemical and biological properties, nanomaterials are widely used in drug delivery systems (DDS). Compared to conventional DDS, nano-DDS can effectively enhance the therapeutic efficacy by improving the pharmacokinetic and pharmacodynamic properties of encapsulated drugs, including drug stability, and achieving targeted drug delivery and controlled drug release due to their special characteristics of size, shape, and material (Yetisgin et al., 2020).

**FIGURE 1:**
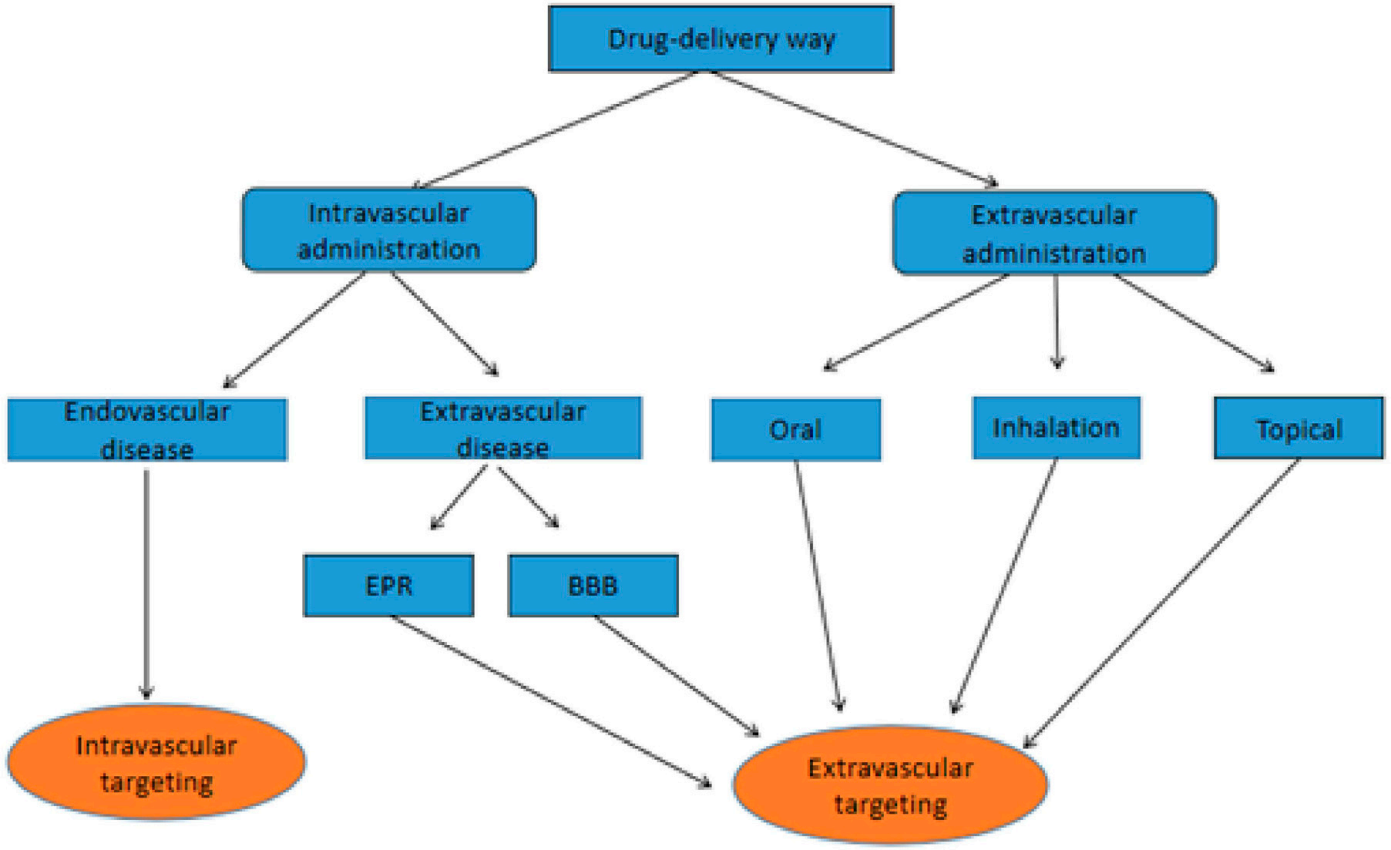
Flowchart of different routes of administration and endovascular targeting vs. extravascular targeting.

Targeted drug delivery refers to successfully targeting therapeutic agents and their primary accumulation at the desired site. Combining therapeutic drugs with nanoparticles and designing suitable targeting pathways is a promising targeted drug delivery method that can deliver many molecules to specific locations in the body (Jahan et al., 2017). To achieve high targeting efficacy, the DDS must be retained in the physiological system for an appropriate time to target specific cells and tissues to release the delivery drug, avoiding its destruction by the immune system (Davis et al., 2008). Nanoparticles can improve the stability and solubility of encapsulated cargo, facilitate transmembrane transport, and prolong cycle times, thereby improving safety and effectiveness (Kou et al., 2018). Nanoparticles can enter the bloodstream through blood vessels and then act at specific sites within the blood vessels to treat intravascular diseases, which is called intravascular drug delivery. Nanoparticles can also cross the endothelium of blood vessels or reach target tissues through local administration such as oral, inhalation, subcutaneous administration, *etc.*, which is called extravascular drug delivery (Figure 1). However, the precise delivery of therapeutic drugs to the target area remains an important issue that needs further investigation to enhance the treatment efficiency of various diseases. In this review, we discuss the design features and therapeutic approaches of different nanomaterials according to the targets of nanomedicines: intravascular and extravascular diseases (Table 1). We also explore some of their limitations and provide an outlook on future directions for targeted drug delivery.

**TABLE 1:** The design of nano-DDS in endovascular and extravascular targeting.

**Targeting type**	**Nanosystem**	**Drugs**	**Targeting moieties**	**External stimuli**	**Key design features**	**Clinical translation**	**References**
Intravascular targeting	VCAM-1–targeting polyelectrolyte micelle	miR-92a inhibitor	Inflamed ECs		Nucleotide-based therapies; polyelectrolyte complex micelles protect the nucleic acids from enzymatic degradation	None	Zhou Z. et al. (2021)
	VHPK-conjugated-pBAE NPs	Anti-miR-712	Inflamed ECs		Overexpression of miR-712 leads to endothelial dysfunction and AS	None	Dosta et al. (2021)
	PEG-b-PLGA copolymer-based NP	CRISPR-Cas9 plasmid DNA	Vascular ECs		NP delivery CRISPR plasmid DNA under the control of the Cdh5 promoter; genome editing in the ECs	None	Zhang X. et al. (2022)
	fAuNPs	DMXAA	Tumor vessels	PTT	DMXAA enhance the local trapping of gold NPs; increased effectiveness of photothermal	None	Hong et al. (2020)
	RGD@HCuS(VA)	VA	ECs	NIR laser	N2 bubbles released by NIR radiation to induce tumor cell necrosis	None	Gao W. et al. (2017)
	Th-Dox-NPs	Thrombin and Dox	Tumor-vessel walls and tumor stroma		Active tumor tissue-targeting ability; cutting of the blood supply by thrombus and inhibiting tumor cell proliferation; synergistic treatment of embolization therapy and chemotherapy	None	Li S. et al. (2020)
	Leukosomes		Inflammatory vascular		Reduced RES uptake; adhesion to inflamed endothelium	None	Martinez et al. (2018)
	Leukosomes	Rapamycin	Atherosclerotic plaques		High histocompatibility; stable drug release efficiency	None	Boada et al. (2020)
	RBC-membrane-camouflaged polymeric NP		Vascular		Long blood retention time	None	Hu et al. (2011)
	PNP	Rapamycin	Atherosclerotic plaques		Reduce the phagocytosis of NPs by macrophages	None	Song et al. (2019)
	Neutrophil cell-membrane-formed nanovesicles		Vascular inflammation		Nanovesicles can improve the specificity and affinity to the target; reduce the lung infammation and edema	None	Gao et al. (2016)
	H_2_O_2_/PFP phase-change NPs		Thrombolysis	US	Oxygen release under ultrasound application	None	Jiang et al. (2020)
	PHPMR NPs	MnFe_2_O_4_、 HMME、 PFP	ECs	US、SDT	High ROS production efficacy under US and SDT	None	Yao et al. (2021)
	SWNTs		Immune cells	PAI	Utilized PAI-active nanomaterial agents assess immune cells migration into tumors	None	Gifani et al. (2021)
Extravascular targeting	Dox/F127&P123-Tf	Dox	Tumor cells		DOX/F127&P123-Tf can inhibit cell migration and change the cell cycle	None	Soe et al. (2019)
	FDMCA	MIP-3β plasmid	Immune cells in tumor		Targeted gene delivery system to polarize macrophages toward M1, inhibiting tumor growth	None	He et al. (2020)
	^64^Cu-GE11 PMNPs		HCT116 colon cancer cells		^64^Cu-GE11 PMNPs have a desirable prolonged residence time in the blood pool and a significant uptake in EGFR-expressing tumor tissue	None	Paiva et al. (2020)
	TLS11a-LB@TATp-MSN/DOX	DOX	Nuclei of H22 cells		NPs contained liver cancer-specific aptamer TLS11a and nuclear localization peptide TATp to locate the nucleus	None	Ding et al. (2020)
	PM@CDDP/SNP	Cisplatin and sodium nitroprusside	Tumor		PMCS selectively induced ONOO− generation after a cascade responding high levels of NOXs and GSH in tumors	None	Chen Y. et al. (2021)
	Col-TNPs		ECM		Col-TNPs deliver collagenase to degrade collagen in tumors, enhancing the intracellular transcytosis of TNPs	None	Wang et al. (2022)
	siFAK + CRISPR-PD-L1-LNPs	FAK siRNA, Cas9 mRNA and sgRNA	TME		Enhanced gene editing efficiency by altering the mechanical properties of the tumor microenvironment	None	Zhang et al. (2022a)
	PLP–D–R	DOX and antiplatelet antibody R300	Tumor vessels		MMP2 can promote the release of R300 in NPs, which can consume platelets and improve tumor vascular permeability	None	Li S. et al. (2017)
	GE11-PDA-Pt@USPIOs	Cisplatin, poly dopamine	EGFR-positive tumor cells	MRI/PAI	The thick PAA coating allows highly efficient cisplatin; the thin PDA coating endows the particles with photo-thermal properties	None	Yang et al. (2019)
	PEG-TK-DOX	DOX,PhA	The tumor	PDT	The production of exogenous ROS during PDT, leading to a further ROS cascade that accelerates the release of DOX and PhA in NPs	None	Kim et al. (2020)
	IT-TQF NPs	IT-TQF	The tumor	PTT	First phototheranostic agent; outstanding optical and photothermal properties	None	Lou et al. (2022)
	MMP sensitive liposomes coated with PEG		ECM of the tumor	US	The combined use of US and microbubbles can increase the penetration depth of liposomes into the extracellular matrix	None	Olsman et al. (2020)
	FCNPs		Vessels	pHIFU	pHIFU can reduce irreversible thermal effects by reducing the average intensity of US	None	You et al. (2017)
	^89^Zr-NRep	DOX	Tumor vessels	EP	^89^Zr-NRep can be used to monitor the effects of electroporation and predict RE-mediated uptake of nanoparticle therapeutics *in vivo*	None	Srimathveeravalli et al. (2018)
	pHA-αPDL1	α-PDL1	Brain and the glioma		pHA-αPD-L1 can activate T cell immune response and relieve immunosuppressive TME	None	Guo et al. (2020)
	TfR-targeting liposomes	Cisplatin	ECs	FUS	The use of FUS in combination with intravascular MBs can increase in BBB permeability	None	Olsman et al. (2021)
	PLGA-PEG NPs		Intestinal epithelium		Albumin engineered enhanced receptor binding and transcellular transport of NPs	None	Azevedo et al. (2020)
	MOF-Gel NPs	Exendin-4	Intestinal epithelium		Neutral PH encouraged sodium bicarbonate and citric acid to dissolve and reacted with CO2, thus facilitating the release of NPs from the capsules	None	Zhou Y. et al. (2021)
	Polypeptide/siRNA polyplexes	siRNAs	Mucus layer and cell membrane		Fluorination of the cationic polypeptides potentiated the mucus-penetration capability; first example of transmucus gene delivery by using the fluorination approach	None	Ge et al. (2020)
	Tf-AMQ NPs	AQ	NSCLC cell		Cytotoxicity studies shown reduction in IC50 values with Tf-AMQ NPs; AQ’s autophagy inhibition ability increased in Tf-AMQ NPs	None	Parvathaneni et al. (2021)
	AG@MSNs-PAA	AG	OA		PAA degraded in an acidic environment, releasing AG to restore IL-1β-induced chondrocyte apoptosis	None	He et al. (2021)
	BNPs	EB	Peritoneal carcinomatosis		The BNPs with abdominal tissue extended the retention after i.p. injection; EB can against multiple PTX-sensitive *in vitro*	None	Deng et al. (2016)
		EB	Glioblastoma		NPs with ‘stealth’ properties can avoid internalization by all cell types	None	Song et al. (2017)
		CPT	SCC tumor cell and matrix proteins		The encapsulation of CPT within BNPs enhanced the delivery of CPT, ; chemotherapy may be beneficial in immune suppression	None	Hu et al. (2021)
		EVG	Vaginal lumen		BNPs can penetrate mucus and once in contact with epithelial cells or leukocytes, they become immobilized and are retained for long periods	None	Mohideen et al. (2017)
	DES-MSNs		Skin penetration		DES-MSNs could drive the MSNs to penetrate across the entire skin via a “Drag” effect; transdermal delivery of the MSNs into blood circulation	None	Zhao et al. (2022)
	CAT-TCPP/FCS NPs	CAT-TCPP	Intratumoral	SDT	Non-invasive excitation of PS in orthotropic bladder; tumor hypoxia amelioration triggered by the O_2_ production of tumoral endogenous H_2_O_2_ catalase	None	Li G. et al. (2020)
	P/B-COS NPs	BUD	Myocardium	RF	Local high temperature in RF promoted local rupture of NPs, releasing BUD	None	Liu et al. (2022)

## 2 Intravascular targeting nanoparticles

Within the past 10 years, the DDS targeted to intravascular diseases has been widely used as a prospective treatment method, including cancers and cardiovascular diseases. Adult blood vessels cover a surface area of 43,000 to 75,000 square feet. However, only a small part of this surface area is affected by vascular disease, showing local inflammation (Davies et al., 2013; Zhou et al., 2014; Gimbrone and García-Cardeña, 2016). Reduction of vascular inflammation by systemic drug therapy usually increases pathogen infection and delays tissue repair (Ridker et al., 2017). Therefore, nanomaterials can be used as vascular-targeted carriers for intravascular drug delivery, reducing the side effects caused by systemic drugs through local targeting. The altered local environment caused by intravascular inflammation can provide ideas for the design of nanoparticles. Increased inflammatory factors, immune cells, and receptors on immune cell membranes all become targets for nanoparticles. Nanoparticles are often modified for intravascular targeting, such as binding peptides, antibodies, and other ligands, coating the surface with cell membranes, and binding the nanoparticles to external physical stimuli. These modification techniques are also commonly combined to enhance the vascular targeting of nanoparticles.

### 2.1 Surface modification and active targeting

The surface of nanocarriers is specifically modified to enter the vascular system and bind to markers on the vascular wall to release the loaded drug for local therapeutic effects (Kelley et al., 2016). This disease-site-specific release increases the drug efficacy and reduces side effects due to systemic administration. It has been reported that the ability to target ligands to nanomaterials enables cell specificity of therapeutic genes/drugs (Arias et al., 2018; Yang et al., 2018; Aizik et al., 2020; Lockhart et al., 2021). Additionally, the vascular endothelium is an effective target for vascular-targeted therapy. The vascular endothelium comprises endothelial cells and vascular lumen and significantly participates in tissue fluid balance and vascular homeostasis (Aird, 2008; Rafii et al., 2016).

Endothelial dysfunction is the root cause of many diseases such as atherosclerosis, major cardiovascular diseases (peripheral artery disease, coronary artery disease, stroke, and myocardial infarction) (Libby et al., 2002; Hansson, 2005), sepsis, acute respiratory distress syndrome (Lee and Slutsky, 2010; Matthay et al., 2012) and COVID-19 respiratory distress (Matthay et al., 2012). Locally disrupted blood flow can significantly enhance the expression of vascular cell adhesion molecule 1 (VCAM-1). Evident high and low VCAM-1 levels are also observed in inflamed and healthy endothelium, respectively (Davies et al., 1993; Nakashima et al., 1998). Zhou et al. designed a VCAM1-targeting polyelectrolyte complex micelles targeting inflammatory vascular endothelium, which could efficiently deliver therapeutic nucleotides to inflammatory endothelial cells (Zhou Z. et al., 2021), similar to (Dosta et al., 2021).

Gene editing of endothelial cells (ECs) is also a promising therapeutic approach. Recently, a novel delivery system using genome editing was developed by Zhang X. et al. (2022) for vascular Ecs. Exogenous genes can be introduced to inhibit vascular injury and/or promote vascular repair, and gene editing systems can be delivered to correct gene mutations and turn specific genes on or off. The nanoparticles carrying CRISPR-Cas9 gene editing plasmids induced efficient gene editing in Ecs of mouse vasculatures, as well as peripheral blood vessels, aorta, heart, and lung. Consistent with the phenotype of genetic knockout mice, significantly decreased levels (80%) of the protein encoded by PI3KCG in EC as selectively observed using immunofluorescent staining and Western blotting. This delivery system represents an important breakthrough in the treatment of diseases caused by endothelial dysfunction, as well as cardiovascular research. Local inflammation usually occurs in intravascular diseases such as atherosclerosis. Inflammation is a body’s natural and necessary response to tissue damage. The enhanced EPR effect occurs at inflamed vessels (Tu et al., 2022), perhaps this is one of the reasons for facilitating the passage of nanoparticles through the vascular endothelium to reach the subendothelial lesions. The High levels of reactive oxygen species (ROS) and low pH in the inflammatory environment can also serve as conditions to trigger the response of nanomaterials (Tu et al., 2022).

However, in the cardiovascular system, the transport of drug-loaded nanocarriers to the target site occurs under dynamic conditions, such as blood flow and its related hemodynamics (Fromen et al., 2016; Ta et al., 2018). Shear stress is also associated with blood flow and plays an important role in the cardiovascular system. Changes in the shear pattern and magnitude are commonly found in cardiovascular and bleeding diseases (Wang Y. et al., 2021). This physiological resistance should be considered for active modification of vascular-targeted carriers. Several studies have recently investigated targeted functionalized nanoparticles under shear stress conditions *in vitro* to address this challenge (Martínez-Jothar et al., 2020; Zukerman et al., 2020). Zukerman et al. used *in vitro* microfluidic perfusion models to investigate the adhesion of nanoparticles to the endothelium of inflammatory blood vessels under pathological high shear conditions (>70 dyne/cm^2^). Their results suggested that, under high shear stress conditions, nanoparticles can act as activated endothelial ligands, such as anti-ICAM-1 antibody, E-selectin-binding peptide (Esbp), or their combination and selectively target activated endothelial cells (Zukerman et al., 2020).

The shear stress of blood flow can act as resistance and a driving force for drug release from nanomaterials. Shear-sensitive nanomaterial drug delivery systems have been increasingly investigated, as well as their therapeutic potential to treat hemorrhages and cardiovascular diseases. As major drug carriers triggered by shear stress, shear-deformable nanoparticles can release the drug according to their physical deformation. Recently, using spherical liposomes, Molloy et al. tested the drug release induced by shear. They achieved site-directed delivery of the antiplatelet drug eptifibatide using shear-sensitive phosphatidylcholine (PC)-based nanocapsules to inhibit platelet aggregation at stenotic sites. Moreover, *in vitro* experiments have shown that, under shearing activation, anti-thrombotic drugs can be delivered by these nanoparticles along with the flowing of liposomes-contained whole blood over collagen matrix at different shear rates. Additionally, *in vivo* studies with mice have found that the drug carrier can target high-shear areas. Decreased thrombus formation without prolonging systemic bleeding was also observed (Molloy et al., 2017).

**FIGURE 2:**
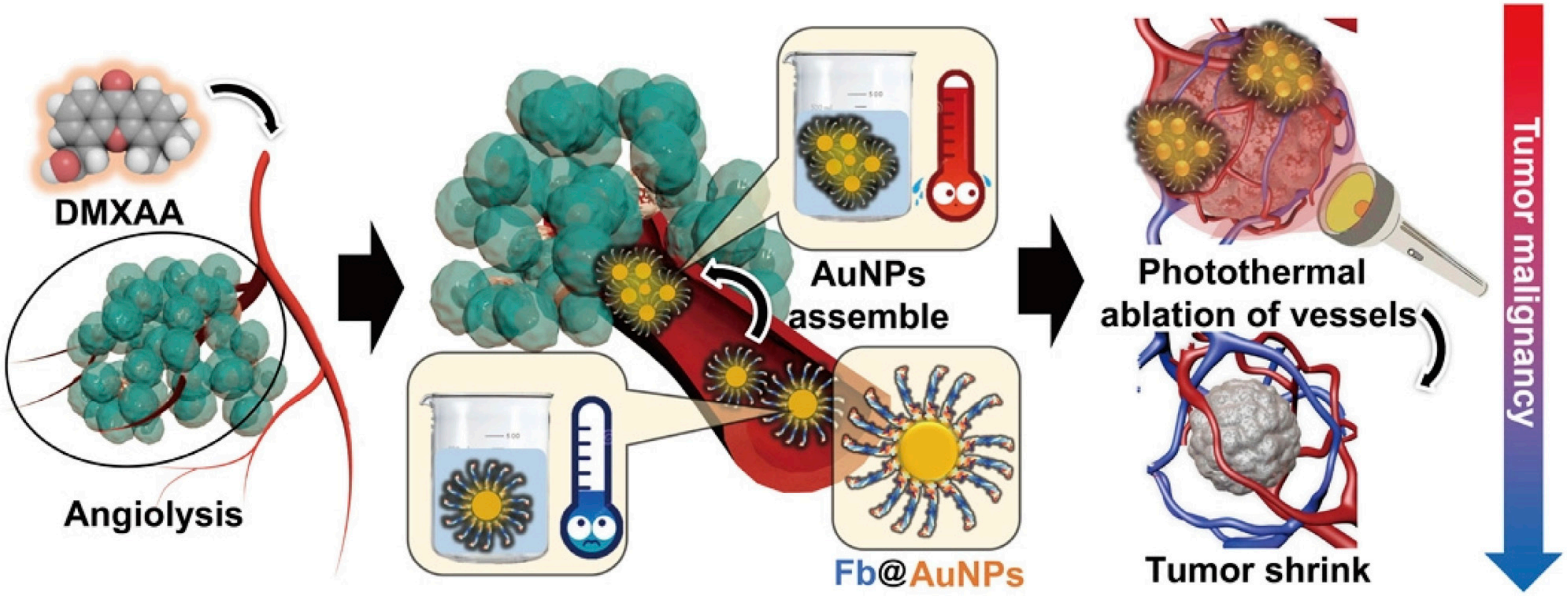
The DMXAA treatment disrupts tumor vessels and subsequently induces AuNPs aggregation in the tumor vessels. Afterward, near-infrared irradiation is applied to achieve the combination of VDAs with PTT for peTVD and photothermally ablate the tumor vascular. Fb, fibrinogen (Hong et al., 2020). Copyright^©^ 2020, AAAS.

Targeting tumor vasculature can also be a potentially effective therapeutic strategy to inhibit primary tumors and metastasis. Angiogenesis is critical for tumor cell proliferation, invasion, and metastatic spread during solid tumor development. Angiogenesis involves forming and remodeling new blood vessels that supply tumors with oxygen, nutrients, and growth factors (Wagner et al., 2015). It is also closely related to tumor development and distant metastasis. Therefore, inhibiting tumor angiogenesis has become a new strategy for tumor treatment. Binding targeted ligands (e.g., antibodies or peptides) on the surface of nanomaterials allows them to bind to receptors specifically expressed in tumor vessels but not in healthy tissue. Active targeting results in higher local intravascular concentrations of nanoparticles and increased retention time in leak-free tumor tissues compared to non-targeted forms (Golombek et al., 2018). DMXAA (5,6-dimethylxanthenone-4-acetic acid ASA404) is a typical small-molecule tumor vascular disrupting agent (VDA) in current clinical trials. It can selectively disrupt tumor blood supply, destroy the tumor vasculature, and target the tumor vascular endothelium, resulting in tumor cell ischemic necrosis. DMXAA can also induce the activation of coagulation cascade reaction and rupture of tumor vessels, enhancing the assembly of soluble fibrinogen into insoluble gel-like fibrin in the tumor vessels. Additionally, Hong et al. (2020) prepared fibronectin-conjugated AuNPs (fAuNPs), which were used with DMXAA for tumor vascular disruption therapy (Figure 2). Guided by DMXAA, fAuNPs aggregated at tumor vessels, and further photothermal treatment was performed to enhance the destruction of tumor vessels. Meanwhile, Gao et al. designed a “nanobomb” RGD@HCuS (VA) targeting tumor vascular endothelium. With the *in vitro* irradiation of the NIR laser, the elevated local temperature triggered the rapid release of N_2_ bubbles from nanoparticles. These N_2_ bubbles exploded instantaneously, destroying the neovascularization and inducing necrosis of surrounding tumor cells. Due to the modification of RGD peptide on the surface of nanoparticles, it binds to αvβ3 receptors on the vascular endothelium to achieve precise destruction of tumor neovascularization without damaging normal tissues (Gao W. et al., 2017). Tumor vascular infarction can also inhibit tumor angiogenesis. Researchers have conducted numerous studies on tumor vascular infarction, which have yielded excellent tumor treatment effects in animal experiments (Shi et al., 2018; Li S. et al., 2020). A recent study reported combining tumor embolization therapy and chemotherapy with chitosan-based polymeric nanoparticles (Th-Dox-NPs). The nanoparticles were modified with CREKA peptides targeting fibrin–fibronectin complexes overexpressed in tumor vessel walls and stroma. The chemotherapeutic agent doxorubicin (Dox) and the coagulation-inducing protease thrombin were also encapsulated. The authors confirmed the ability of Th-Dox-NPs to induce tumor vascular thrombosis due to the action of thrombin using animal experiments and histological analysis. Compared to nanoparticles delivering only thrombin or Dox, Th-Dox-NPs significantly inhibited tumor growth and recurrence after injection and did not cause somatic toxicity (Li S. et al., 2020). This study provided a paradigm for vascular embolization-based tumor therapy by enabling payload and protease co-delivery through biodegradable tumor-targeting nanoparticles that cause embolization of tumor blood vessels to achieve effective tumor inhibition. Usually, tumor infarction can be acutely induced, and its effects can occur within a few hours (Jahanban-Esfahlan et al., 2017).

### 2.2 Biomimetic modification and homologous targeting

Once nanomaterials enter the human body, they encounter a complex physiological environment and immune defense system that actively identifies and removes substances unfamiliar to the human body (Moyano et al., 2016; Bourquin et al., 2018). One of the major obstacles is that nanoparticles are absorbed and cleared by the reticuloendothelial system before reaching their targets, to the extent that few nanoparticles loaded with targeting ligands make it through Phase III clinical trials (Hu et al., 2017; Mo et al., 2018; Gao et al., 2020). Therefore, developing biomimetic nano-delivery systems that effectively evade clearance by immune capture and have better targeting capabilities for long-term blood circulation is crucial (Chen et al., 2019). In particular, the use of cell membrane-modified nanoparticles of inflammatory cells is not only effective in avoiding immune capture, but also in achieving better aggregation of inflammatory regions. Such cell-derived nanoparticles can transfer phospholipid bilayers and membrane proteins from cell membranes to nanocarriers, which enables better biocompatibility and targeting of nanoparticles under natural cell membrane camouflage (Fang et al., 2014; Narain et al., 2017).

**FIGURE 3:**
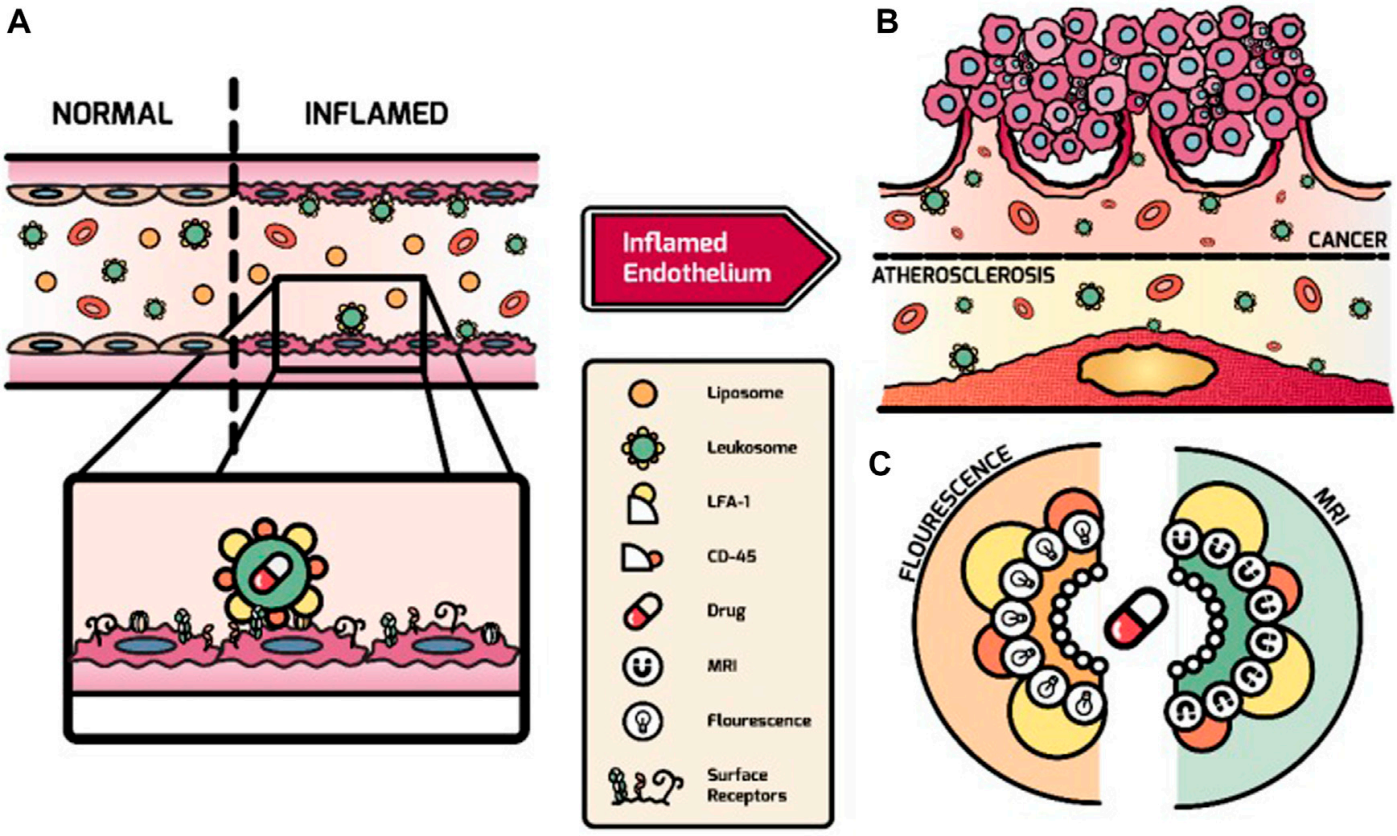
Overview of the targeting process of leukosomes to activated endothelium. **(A)** Mediated by LFA-1 and CD45, leukosomes can selectively bind to activated endothelium after systemic administration. **(B)** Leukosomes experience effective targeting of atherosclerosis and cancer-related inflammation. **(C)** The imaging modalities (e.g., fluorescence dyes and Gd) and therapeutics can be incorporated by leukosomes with their versatility (Martinez et al., 2018). Copyright^©^ 2018, Ivyspring.

Biomimetic nanoparticles covering immune cell membranes, such as leukocytes and macrophages, are widely used to treat vascular injury, inflammation, and cancer (Wang et al., 2015; Martinez et al., 2018; Boada et al., 2020; Gao et al., 2020; Wu et al., 2020; Liu et al., 2021). Leukocyte and macrophage membranes are the most attractive materials for biomimetic DDS. As the main cellular components of innate immunity, leukocytes and macrophages can be better recruited and accumulated into vascular inflammatory cells (Vemula et al., 2010; Amengual and Barrett, 2019). Leukocytes can be rapidly activated in vascular inflammation and migrate to the inflammation site (Dong et al., 2017; Chu et al., 2018). The molecules expressed in inflammatory vascular endothelium, such as selectins, ICAM-1, and VCAM-1, significantly promote transendothelial extravasation, adhesion, and recruitment of circulating leukocytes to the vessel wall (Pober and Sessa, 2007; Galvani et al., 2015; Jin et al., 2018). Martinez et al. (2018) studied leukocyte-based biomimetic nanoparticles called leckosomes (Figure 3). The authors assembled leukocyte phospholipids and membrane protein binding into leckosomes, which accumulated in inflammatory vascular lesions fourfold more than controls after administration to mice with atherosclerotic plaques. They also demonstrated that the targeting effect of leckosomes was due to the expression of lymphatic CD45 and function-associated antigen-1 (LFA-1) on the leukocyte membrane. Due to the lipid properties of leckosomes, they can be used as a novel therapeutic agent loaded with therapeutic drugs with different physical properties. Furthermore, Boada et al. used bionic drug carrier leckosomes loaded with rapamycin for atherosclerosis treatment, which could inhibit the proliferation of macrophages in the aorta and slow down atherosclerosis (Boada et al., 2020).

Erythrocytes are considered long-term circulatory transport carriers *in vivo*. Due to their highly hydrophilic properties, the stability of the nanoparticles encapsulated by the erythrocyte membrane can be enhanced with polysaccharides on the erythrocyte membrane surface (Yang L. et al., 2020). As drug carriers, erythrocytes can prolong the half-life of drugs and reduce adverse reactions and immunogenicity (Luk et al., 2014; Zhang et al., 2017; Alapan et al., 2018). Additionally, erythrocyte membranes express CD47 transmembrane protein, which blocks macrophage uptake by selectively binding to SIRPα expressed by macrophages (Wang et al., 2020b). Thus, nanoparticles disguised by erythrocyte membranes might prolong the time in blood circulation and enhance their levels in the target site. Hu et al. first reported a natural erythrocyte membrane-encapsulated poly (lactic-co-glycolic acid) (PLGA) nanoparticle that significantly prolonged the *in vivo* circulation time compared to only PLGA and PEGylated PLGA (Hu et al., 2011). Studies have also reported that nanoparticles coated with erythrocyte membranes have significant therapeutic effects in treating thrombosis, atherosclerosis, sepsis, and other vascular diseases (Wang Y. et al., 2019; Ben-Akiva et al., 2020; Zhao et al., 2020; Liang et al., 2022).

Besides other proteins such as CD55 and CD59, the platelet membrane surface expresses CD47 to protect them from the absorption of macrophages and enable long-lasting circulation in the vasculature (Hu C. M. et al., 2015; Hu Q. et al., 2015). Studies have demonstrated that P-selectin and CD40 ligands expressed on the surface of erythrocyte membranes can regulate many disease processes, especially inflammation and tumors (Borsig et al., 2001; Sprague et al., 2007). According to the characteristics of aggregation and adhesion and the physiological function of platelets in inflammation, the targeting of atherosclerotic disease has been achieved by platelet membrane-encapsulated nanoparticles (Kunde and Wairkar, 2021). This idea is also supported by the usage of nanoparticles coated with platelet membranes in the imaging of atherosclerotic plaque (Wei et al., 2018). The ability of nanoparticles to interact with Ecs has been used to develop platelet membrane camouflaged nanoparticles to treat atherosclerosis (Wei et al., 2018; Song et al., 2019; Chen L. et al., 2022). For example, Song’s team developed platelet membrane-encapsulated PLGA nanoparticles loaded with anti-atherosclerotic rapamycin. The *in vitro* adhesion experiments were analyzed by flow cytometry and fluorescence imaging. These platelet membrane-encapsulated nanoparticles bound 8.34 times more fluorescently to collagen and 9.61 times to fibronectin than the control. Additionally, compared to the control, the biomimetic nanoparticles exhibited significantly high targeting activity *in vivo* to atherosclerotic plaques (4.98 times), indicating the long-lasting circulation and effective adhesion of biomimetic nanoparticles to plaques *in vivo*. After *in vivo* treatment in mice, atherosclerotic plaque lesions in the aorta were significantly reduced by 14.5% ± 2.9% compared to other nanoparticles and free rapamycin (Song et al., 2019).

Extracellular vesicles (EVs), produced and released by cells such as neutrophils and platelets, can specifically target inflammation and tissue sites (Shah et al., 2018). Gao et al. (2016) designed a DDS based on a nanovesicle with a nanobubble core formed by cell membranes produced by activated neutrophils. The EVs derived from neutrophils could interact with Ecs by binding to the ICAM-1 (Kolaczkowska and Kubes, 2013). The EVs derived from platelet are significantly involved in thrombosis. The coagulation activity of these EVs is 50–100 times greater than activated platelets in circulation (Yin et al., 2015). Meanwhile, injecting EVs sourced from platelets through the tail vein in tail-bleeding model mice could induce hemostasis. Platelet treatment reduced bleeding by 45% compared to controls, while treatment with EVs isolated from platelet decreased bleeding by 62% (Miyazawa et al., 2019).

Cell-derived biomimetic nano-DDS cleverly combine the advantages of nanoparticles and natural biofilms to achieve some remarkable achievements in the treatment of vascular diseases, allowing better biocompatibility, longer *in vivo* circulation time, and targeting of inflammatory sites. However, many challenges remain. For example, compared to ordinary nanoparticles, highly synthetic techniques and complex procedures are required for biomimetic nanoparticle preparation using cell membranes. Maintaining nanoparticles’ physicochemical and biological properties during synthesis is crucial (Molinaro et al., 2018). Zinger et al. studied the effects of different parameters in the synthesis process of biomimetic nanoparticles on their physicochemistry and biomimetic properties and targeting (Zinger et al., 2021). During the extraction and encapsulation of cell membranes, maintaining the surface biological factors’ function and integrity of the cell membrane are important to producing biomimetic nanoparticles on a large scale. Detachment of cell membrane structure or premature drug release during nanomedicine therapy can also lead to reduced targeting and efficacy (Yang L. et al., 2020).

### 2.3 Physical triggering strategies

The combination of nanoparticles with physical triggering strategies such as ultrasound (US), magnetic fields, and light allows for precise targeting of nanomedicines within the vasculature, improving targeting and controlling drug release. Microbubbles are commonly used clinically as ultrasound contrast agents with a shell composed of lipids, proteins, or polymers and an interior encased in air or perfluorocarbons. Under ultrasound at a certain frequency, microbubbles cavitate; that is, they oscillate, expand, and implode (Weiss et al., 2013). Nanoparticles can also be coated with liquid perfluorocarbon, and the core perfluorocarbon can vaporize to the gas phase upon activation by ultrasonic energy, known as acoustic droplet vaporization (ADV), followed by the nanoparticles growing in size to form microbubbles, which further burst under the action of ultrasound to release the drug locally (Hou et al., 2022). Additionally, ultrasound-irradiated phase-change nanoparticles have great potential for vascular thrombolysis. Jiang et al. (2020) designed an extracorporeal artificial circulation system to simulate thromboembolism in vascular circulation *in vitro*. H2O2/PFP phase transition nanoparticles could produce sustained ultrasound-targeted microbubble destruction (UTMD) with a durable cavitation effect and high thrombolytic efficiency. Microbubble targeting to thrombus sites is time-sensitive, and ultrasound-triggered microbubbles do not achieve maximum thrombolytic efficiency. Therefore, nanoparticles that can actively target and bind the thrombus are usually designed to increase the efficiency of ultrasonic thrombolysis (Zhong et al., 2019; Yang A. et al., 2020; Ma et al., 2020; Gao et al., 2021).

Furthermore, exposure of magnetic microbubbles to magnetic fields can synergistically improve the efficiency of ultrasound thrombolysis and limit long-term exposure (de Saint Victor et al., 2019; Zhang et al., 2019). Enhanced cavitation effects are observed when magnets are placed at thrombus sites. Thus, magnetic nanoparticles (MagNP) comprise a new tool for intravascular targeted drug delivery of nano-DDS. At the same time, as a contrast agent, the superparamagnetic iron oxide nanoparticles can be used to diagnose cardiovascular diseases by magnetic resonance imaging (MRI) (Zhang S. et al., 2020). For example, researchers have designed PHPMR NPs for the non-invasive treatment of atherosclerotic plaque neovascularization. It encapsulates manganese ferrite (MnFe2O4), hematoporphyrin monomethyl ether (HMME), and perfluoropentane (PFP), stabilized by a poly (lactic acid-glycolic acid) (PLGA) shell and bound to an anti-VEGFR-2 antibody. MRI, photoacoustic, and ultrasound imaging were performed. PHPMR NP-mediated sonodynamic therapy (SDT) inhibited neovascularization by generating reactive oxygen species (ROS) to promote apoptosis (Yao et al., 2021). Vascular visualization is important in the diagnosis and treatment of cardiovascular diseases. Researchers have recently developed a carbon nanotube imaging system in which macrophages and monocytes can absorb nanoparticles in atherosclerosis, and photoacoustic imaging *in vitro* can accurately locate and visualize plaques, providing a new approach to the clinical diagnosis of relevant cardiovascular diseases (Gifani et al., 2021). Although researchers have conducted various studies on magnetic DDS, their clinical application remains limited because of the safety of magnetic nanoparticles and because there is no clinically approved medical device to apply a magnetic field to nanoparticles in blood vessels (Sun et al., 2020; Zenych et al., 2020).

## 3 Extravascular targeting nanoparticles

Additionally, to target intravascular diseases through blood vessels, nanoparticles can target tumor and inflammatory tissues across the vascular endothelium by their enhanced permeability and retention (EPR) effect or neurological diseases by crossing the blood-brain barrier (BBB). Besides intravenous nanoparticles, they can be administered by inhalation and oral and topical administration.

### 3.1 Binding targets across the vascular endothelium

#### 3.1.1 EPR

Tumor overgrowth results in neovascularization with unusually wide fenestrations and dysplasia of lymphatic vessels in solid tumors, allowing preferential accumulation of nanoparticles in tumors, a phenomenon known as the EPR effect (Maeda et al., 2013; Nakamura et al., 2016), which Matsumura and Maeda first proposed in 1986 (Matsumura and Maeda, 1986). Besides tumors, the EPR effect is used in diseases such as infection, inflammation, and atherosclerosis (Gao et al., 2019; Clausen et al., 2020; Tu et al., 2022). EPR effects are frequently observed in the vascular and tumor sites of inflamed tissues. In general, nanoparticles of 20–200 nm diameter are best suited for passive targeting of drugs to inflamed tissue (Lundy et al., 2016). A recent study in rheumatoid arthritis (RA) showed that 100 nm-sized egg yolk lecithin (EPC) liposomes circulate in mice for up to 12.85 h. The longer the circulation time, the more they accumulate in the inflamed joints (Ren et al., 2019). Endocytosis of nanoparticles by immune cells can be influenced by the shape and surface charge of the nanoparticles (Tu et al., 2022). In this section, we focused on applying EPR effects in tumors. Understanding the EPR effect provided a basis for developing anti-tumor macromolecular drugs and brought hope to nanomedicine. Therapeutic agents, such as gemcitabine, cisplatin, or doxorubicin, are low molecular weight drugs (typically less than 1000 Da) with poor pharmacokinetics and biodistribution (de Jonge and Verweij, 2006). Advances in nanotechnology have led to the development of many DDS, improving the accumulation of agents for chemotherapy at target sites and their biodistribution. By increasing the size of systemically administered drugs to a diameter of about 100 nm, renal excretion can be reduced (considering a renal clearance threshold of about 40,000 Da), blood half-life can be prolonged, and its accumulation in target tissues can be improved (Golombek et al., 2018).

Anti-tumor nanomedicines, including Doxil, Marqibo, and Abraxane, have been successfully used in clinical practice (Jain and Stylianopoulos, 2010; Barenholz, 2012; Silverman and Deitcher, 2013). These nanomedicines use the EPR effect to improve the pharmacokinetics of chemotherapeutic drugs and reduce systemic side effects. Encapsulation of doxorubicin into liposomes (Doxil) increases the plasma half-life of the free drug from 5 to 10 min to 2–3 days (Gabizon et al., 1994). Doxil, the first FDA-approved nanomedicine, has 100 nm and uses the stealthy polymer polyethylene glycol (PEG) for surface modification to reduce the aggregation and opsonization of plasma proteins, extending its circulating half-life in the blood (Barenholz, 2012; Suk et al., 2016). Doxil leads to 4–16 times higher drug concentrations in malignant effusions than free doxorubicin, a phenomenon demonstrating the EPR effect. Meanwhile, Doxil has been approved for treating ovarian cancer, breast cancer, and multiple myeloma, among other diseases (Min et al., 2015). However, the nanomedicines mentioned above are not good enough to prolong patient survival because nanoparticle delivery using the EPR effect is also hampered by the tumor microenvironment, such as the stromal compartment composed of extracellular matrix (ECM) and mesenchymal cells, interstitial fluid pressure (IFP), and poor blood flow. Collagen, fibronectin, and hyaluronic acid in the ECM form a barrier outside tumor cells that promotes tumor growth, prevents nanodrugs from penetrating deeply into the tumor mesenchyme from blood vessels, and strongly affects the accumulation of nanodrugs in tumors (Yokoi et al., 2014; Nissen et al., 2019). Due to the increased permeability of tumor blood vessels, leakage of substances from the plasma through the blood vessels leads to increased interstitial fluid pressure and the lack of a normal lymphatic drainage system, creating high interstitial fluid pressure impairs the penetration and accumulation of nanodrugs (Gao X. et al., 2017; Glicksman et al., 2017). Previous studies have demonstrated the effects of interstitial fluid pressure on tumor size using tumor models (Ruan et al., 2018). Due to the specificity of tumor neovascularization, blood vessels have impaired perfusion and slow blood flow compared to normal tissues (Hori et al., 2000). However, with the in-depth study of the EPR effect, scientists have increasingly recognized its high heterogeneity. One reason for this is the variability between experimental mouse models and patients. Also, tumors of the same origin vary across patients or periods (Tanaka et al., 2017; Fang et al., 2020).

In summary, the EPR effect alone cannot effectively and completely eliminate tumor cells using nanomedicines. Therefore, modification of the nanoparticle surface with molecular markers and external physical stimuli might be effective “EPR enhancers.” Next, we discuss the mechanisms and research progress of these two emerging strategies.

##### 3.1.1.1 EPR combined with molecular markers enables nanoparticle targeting

Modifying nanoparticles using molecular markers can enhance the active targeting ability of nanoparticles, increase cytotoxicity against tumors and reduce systemic side effects (Table 2). In other words, nanoparticle interactions with target cell surface molecules, such as ligand-receptor, enzyme-substrate or antibody-antigen-mediated interactions, are utilized (Nag and Delehanty, 2019). Many targets are overexpressed in tumor cells, making them potential targets for nanoparticles. Transferrin receptors are overexpressed on the surface of most tumor cells. Conversely, they are underexpressed in normal cells. Moreover, transferrin-modified nanoparticles can enhance drug delivery efficiency in tumor cells (Soe et al., 2019; Bellini et al., 2020). Transferrin-based nanotherapeutic probes have also been developed to achieve early tumor diagnosis through active targeting (Jiang et al., 2022). Widespread upregulation of folate receptor expression in many cancers has also been demonstrated, making it an ideal target for nanomedicines (Wang et al., 2020a; He et al., 2020; Scaranti et al., 2020). Additionally, the epidermal growth factor receptor (EGFR), which belongs to the ErbB family of receptor tyrosine kinases, is overexpressed in various human cancers, becoming a target for cancer therapies even used in clinical treatment (Sigismund et al., 2018). In a recent study, modification of the surface of the nanoparticles with peptide GE11 was performed to deliver the anti-tumor drugs to tumor sites in a colorectal cancer mouse model. The *in vitro* fluorescence results showed significantly enhanced internalization of the treatment nanoparticles on EGFR-expressing HCT116 colon cancer cells compared to controls (Paiva et al., 2020). Furthermore, Ding et al. (2020) designed the TLS11a-LB@TATp-MSN/DOX nanoparticle, specifically targeting H22 cells using the nuclear localization signal peptide TATp and the aptamer TLS11 as modified nanoparticles. The *in vivo* experiments with tumor-bearing mice demonstrated that the nanoparticles could enrich and target chemotherapeutic drug delivery to the nucleus of hepatocellular carcinoma cells in liver cancer tissues. Nanoparticles can also bind tumor-penetrating peptides (TPPs) on their surface, specifically binding to receptors expressed on tumor endothelial cells and performing transendothelial transport pathways to extravasate nanoparticles from tumor blood actively (Liu et al., 2017). As a peptide with tumor penetrating activity, iRGD (CRGDK/RGPD/EC) can be used for the surface modification of nanoparticles. Many experimental studies on iRGD have confirmed that its modification in iRGD can improve the targeting and retention of nanoparticles in tumors (Lin et al., 2020; Li H. et al., 2021; Chen B. et al., 2022). Immune cells in the tumor microenvironment (TME) also influence the aggregation of nanoparticles. For example, tumor-associated macrophages (TAMs) are key to tumor growth (Mantovani et al., 2017), and targeting macrophages as nanoparticles can be a therapeutic strategy. Two recent studies have successfully inhibited the recruitment of TAMs to tumors by specifically targeting CCR2 on macrophages (Zhang X. et al., 2021; Trac et al., 2021). The uptake of nanoparticles by cells is via phagocytic or non-phagocytic pathways, such as clathrin and caveolae-mediated endocytosis, resulting in transendothelial and transcellular transport and relatively uniform distribution throughout the tumor (Li Z. et al., 2017; Zhou et al., 2019). Transcytosis is an important active transport process in which biomacromolecules are transported through dense epithelial and endothelial cells. During transcytosis, biomacromolecules are internalized by cells and excreted from the body (Niu et al., 2021). Transcytosis has been proven to be an effective approach to improve the penetration of nanoparticles into tumors (Wang et al., 2022).

**TABLE 2:** Nanoparticles designed for different components of the tumor microenvironment.

**Target site**	**Nanosystem**	**Molecular markers**	**References**
Tumor cells	Dox/F127&P123-Tf	Tf	Soe et al. (2019)
	FDMCA	Folic acid	He et al. (2020)
	^64^Cu-Labeled GE11-Modified Polymeric Micellar NPs	Peptide GE11	Paiva et al. (2020)
	TLS11a-LB@TATp-MSN/DOX	TATp, TLS11a-LB	Ding et al. (2020)
	d-SN38@NPs/iRGD	iRGD	Lin et al. (2020)
	iRGD-PSS@PBAE@JQ1/ORI NPs	iRGD- pss	Chen B. et al. (2022)
	KLAK-MCP-1 micelles	MCP-1 peptide, KLAKLAK peptide	Trac et al. (2021)
ECM in the TME	PM@CDDP/SNP		Chen L. et al. (2021)
	Col-TNPs	Collagenase	Wang et al. (2022)
	HA-sPLGA XNPs	Hyaluronic acid	Wang X. et al. (2021)
Enzymes in the TME	FRRG-DOX NPs	Cathepsin B-cleavable peptide	Shim et al. (2019)
	HEKMs	EGFR-HER2, FRET	Wang Z. et al. (2019)
	PLP–D–R	Antiplatelet antibody R300	Li S. et al. (2017)
	siFAK + CRISPR-PD-L1-LNPs	FAK siRNA	Zhang D. et al. (2022)
	AuNPs-D&H-R&C	RVRRCK, CABT	Xie et al. (2021)

Besides targeting tumor cells, improving the complex ECM in the TME is a prominent strategy to enhance the penetration and retention of nanoparticles in the tumor ECM (Uthaman et al., 2018). The ECM is inextricably linked to tumor cell development, metastasis, and invasion. Key ECM components, such as hyaluronic acid and collagen, form a dense biological barrier that prevents nanomedicines from penetrating deep into tumor cells, resulting in uneven drug delivery (Poltavets et al., 2018; Guo et al., 2021). Many studies have been reported using new materials and strategies to facilitate the penetration of nanomedicines into the ECM. A tumor-specific ONOO^−^ nanogenerator (PM@CDDP/SNP, abbreviated as PMCS) was designed to produce ONOO^−^ and more matrix metalloproteinases in a specific TME, and degrade its dense ECM, enhancing the EPR effect and promoting drug penetration and accumulation deep in the tumor (Chen Y. et al., 2021). Wang et al. (2022) designed nanoparticles modified with collagenase and demonstrated that the endocytosis of nanoparticles could be effectively increased by degrading collagen, indicating that the dense fibrous collagen prevented the penetration of nanomolecules and chemotherapeutic drugs into tumors. Hyaluronic acid binds to the CD44 receptor, upregulated in various cancer cells, which improves ECM targeting and has good therapeutic potential in some cancers (Liu et al., 2018; Choi et al., 2019; Wang X. et al., 2021). Studies targeting hyaluronic acid-modified nanoparticles are important for further nanomedicine development. For example, Wang et al. used fluorescence imaging and showed that DTX-HA-sPLGA XNPs rapidly potentiated aggregation in tumors and extended the elimination half-life to 4.18 h, 18.2 times the free DTX after 8 h of injection in mice (Wang X. et al., 2021). There are also studies using hyaluronidase to eliminate hyaluronic acid from ECM and a phase III clinical trial with pancreatic cancer patients in 2018 (Doherty et al., 2018; Infante et al., 2018). Besides hyaluronidase and collagenase, other enzymes can degrade the ECM. Higashi et al. (2020) prepared PEG pineapple enzyme to study its therapeutic potential in pancreatic cancer. After PEG intravenous injection, bromelain can significantly accumulate in the tumor through the EPR effect and exhibit a strong ability to degrade the ECM. Therefore, reducing the tumor ECM can enhance the penetration of nanodrugs and increase the intra-tumor drug concentration. Furthermore, it has been shown that the degradation of collagen, a key component of ECM, effectively enhances the intracellular transcytosis of nanoparticles (Wang et al., 2022). Recently, researchers have explored the interaction characteristics between nanomedicines and the ECM. Cheng et al. first found that nanoparticles bind tightly to specific lipid components in the ECM, namely, retraction fibers (RFs) and migraction, to inhibit cell migration. This interaction provides a potential therapeutic target and strategy for nanomedicines against tumor metastasis (Cheng et al., 2022).

**FIGURE 4:**
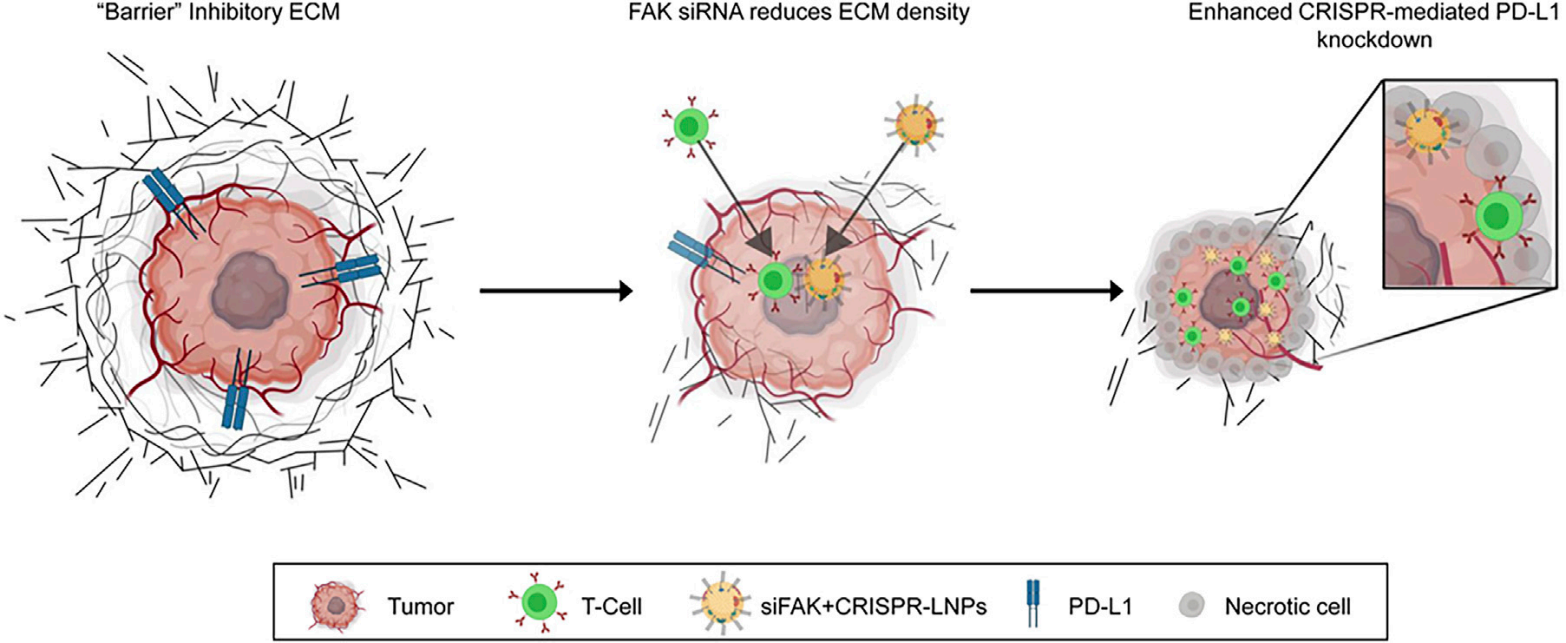
Schematic illustration for targeting the mechanical properties of tumors to open a double-checkpoint blockade (stiff ECM plus immunosuppression) to enable cancer therapy. Proposed model for how dendrimer LNPs encapsulating FAK siRNA, Cas9 mRNA, and targeted sgRNAs enhance penetration into tumors with increased gene editing of PD-L1 for improved cancer therapy (Zhang D. et al., 2022). Copyright^©^ 2022, Springer Nature.

Several specific enzymes in the TME, such as histone B, matrix metalloproteinases, and caspase, can enhance the targeting of nanodrugs and improve therapeutic efficacy (Abumanhal-Masarweh et al., 2019; Shim et al., 2019; Wang Z. et al., 2019). A research team has developed a novel drug delivery system targeting tumor vasculature, a polymer-lipid-peptide-based (PLP) DDS. The PLP was constructed using two portions: a polymeric core nanoparticle and a shell layer. The core nanoparticle was constructed with the biodegradable and biocompatible block copolymer poly (etherimide)-poly (lactic-co-glycolic acid)2 (PEI–(PLGA)2) and loaded with the chemotherapeutic agent DOX and antiplatelet antibody R300. The shell layer contained PEGylated phospholipids, lecithins, and matrix metalloproteinase 2 (MMP2)-cleavable peptides. R300 is specifically released in the tumor on the cleavage of the lipid-peptide shell of the nanoparticles by MMP2, which is commonly overexpressed in tumor vascular endothelia and stroma. R300 induces tumor vascular damage and disrupts the tumor vascular barrier by depleting intra-tumor platelets and promoting massive neutrophil infiltration (Gros et al., 2015; Hillgruber et al., 2015). This treatment can effectively improve tumor vascular permeability, increase the accumulation of drug particles in tumors, promote tumor regression, and inhibit tumor metastasis (Li S. et al., 2017). Additionally, focal adhesion kinase (FAK) activation is related to the generation of ECM stiffness (Seong et al., 2013). Inhibition of FAK activity also enhances the infiltration of CD8^+^ cytotoxic T cells (Serrels et al., 2015; Jiang et al., 2016). For example, Zhang et al. designed a self-assembled nanoparticle LNPs (siFAK + CRISPR-LNPs) targeting FAK in tumor tissues to improve the mechanical characteristics of ECM, enhance drug penetration, and effectively inhibit the growth of cancer cells (Figure 4) (Zhang D. et al., 2022). Also, proprotein convertase furin is a specific target for breast cancer diagnosis and treatment, as it is highly expressed in breast cancer (Jaaks and Bernasconi, 2017; Li et al., 2019; Zhu et al., 2019). A recent study has designed a furin-responsive aggregated nanoplatform to improve drug delivery efficiency (Xie et al., 2021).

##### 3.1.1.2 EPR combined with physical stimulation enables nanoparticle targeting

The ability of nanoparticles to penetrate tumor tissue can be enhanced using physical stimulation. External physical stimulation by laser, ultrasound, radiation, and electric fields can alter the TME and improve the penetration effect of nanomaterials within the tumor.

Radiotherapy (RT) is one of the current clinical treatments for solid tumors, either as monotherapy or combined. RT destroys tumor cells and TME by generating energy and ROS through external radiation exposure but can have some effect on healthy tissue cells (Boateng and Ngwa, 2019). Because of the enhanced expression of vasoactive mediators (e.g., VEGF), induced apoptosis of endothelial cells, and increased tumor vascular permeability, RT can increase the accumulation of nanodrugs in tumor tissues (Ojha et al., 2017). Yang et al. developed a multi-functional theranostic system based on poly acrylic acid-coated ultra-small superparamagnetic iron oxide nanoparticles (PAA@USPIOs), GE11-PDA-Pt@USPIOs. This system was conjugated with GE11 peptide, coated with polydopamine, and loaded with cisplatin. After intravenous injection of nanoparticles, MRI and PA imaging showed that GE11-PDA-Pt@USPIOs efficiently accumulated in tumors. The use of RT further enhanced the antitumor efficacy of chemotherapeutic agents (Yang et al., 2019). Meanwhile, Charest et al. developed a novel nanocomplex by wrapping carboplatin and gold nanoparticles into a liposomal formulation. The dosage of gold nanomaterials and carboplatin equipped in liposomal nanomaterials was the same. The *in vitro* application of RT for combination therapy significantly inhibited tumor growth (Charest et al., 2020). As an adjunct to cancer treatment, heat therapy (HT) has a synergistic effect on chemotherapy and radiotherapy (Dunne et al., 2020). Local hyperthermia of tissues can increase blood flow, change vascular permeability, and promote drug aggregation at the tumor site. Effective synergistic killing of tumor cells by magnetic hyperthermia therapy in combination with chemotherapy has been documented (Song et al., 2020).

**FIGURE 5:**
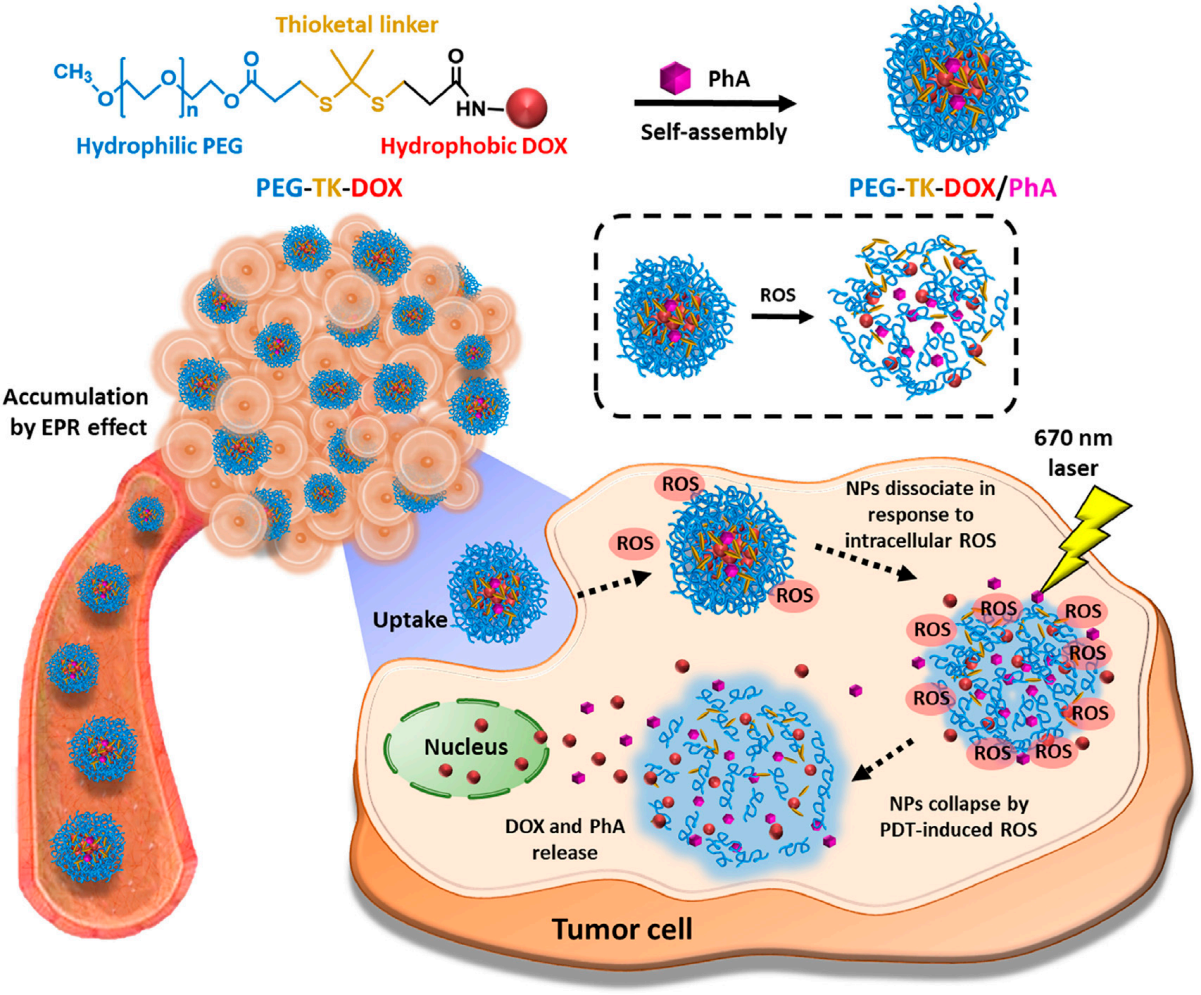
Schematic diagram of the mechanism underlying the action of PEG-TK-DOX/PhA (Kim et al., 2020). Copyright^©^ 2020, Elsevier.

Light is a form of electromagnetic radiation and can be used as a physical trigger strategy. Ultraviolet and visible lights have a shallow penetration depth due to their shorter wavelengths. The opposite is true for near-infrared light. Using light and photosensitizers, two physical triggering strategies, photothermal therapy (PTT), and photodynamic therapy (PDT), can potentially enhance the EPR effect. PDT generates ROS using photosensitizers in combination with specific light wavelengths, leading to cell death (Dhaliwal and Zheng, 2019). PDT induces increased vascular permeability at the tumor site and can improve the EPR effect of nanoparticles after administration (Hare et al., 2017). PDT is a non-invasive treatment, and nanoparticles have been increasingly used for PDT with efficient targeting of tumors (Kim et al., 2020; Paris et al., 2020). Due to the tumor hypoxic microenvironment, many studies have developed nanocarriers capable of performing self-oxygenation to increase the therapeutic efficiency of PDT (Huang et al., 2019; Liu et al., 2020). Kim et al. (2020) developed an adriamycin and photosensitizers-encapsulated nanoparticle system that could effectively respond to ROS (Figure 5). Based on the EPR effect, nanoparticles accumulate in tumors, and endogenous ROS and ROS generated during PDT prompt the nanoparticles to release adriamycin and photosensitizers, and produce more ROS to make nanoparticles accumulate. PTT is a new non-invasive treatment method that uses photothermal agents (PAs) to convert light into heat to kill cells when irradiated by an external light source such as near-infrared light (Zhi et al., 2020). One study reported a novel small molecule NIR-IIb dye IT-TQF with a D-A-D structure encapsulated into DSPE-PEG_2000_ to construct IT-TQF NPs. IT-TQF NPs have good photostability, and their high therapeutic potential for tumor PTT was demonstrated *in vitro* and *in vivo* with photothermal performance tests. The *in vivo* photothermal treatment was performed on a 143B subcutaneous tumor mouse model after intravenous injection of IT-TQF NPs. The tumors were successfully suppressed after photothermal treatment in the IT-TQF NPs combined with the laser treatment group, demonstrating the good photothermal treatment effect of IT-TQF NPs *in vivo* (Lou et al., 2022). However, one limitation of PDT/PTT is that light does not penetrate deep enough into the tissue to allow radical treatment of deep tumors. Therefore, there is an urgent need to develop treatments that can effectively compensate for the shortcomings of PDT/PTT.

Furthermore, ultrasound can significantly promote the EPR effect by enhancing cell membrane permeability. Ultrasound is of great interest due to its easy operation, non-invasive nature, and high tissue penetration depth (Duan et al., 2020). Acoustic perforation comprises the expansion and bursting of gas-filled microbubbles under ultrasound irradiation, which physically disrupts blood vessels to facilitate drug delivery (Duan et al., 2020). Combining the use of ultrasound and therapeutic microbubbles can enhance the EPR effect. For example, Koczera et al. loaded different drug types and targeted poly (n-butyl cyanoacrylate) (PBCA) microbubbles for imaging and drug delivery. Combining the microbubbles with ultrasound allowed the effective passage of drugs through the vessel wall (Koczera et al., 2017). Another study described an ultrasound-mediated liposome nanoparticle-based DDS, in which ultrasound improved nanoparticle accumulation in tumor tissue by increasing penetration of liposomes from the vasculature into the ECM. Tumor sizes were reduced, and survival was prolonged in mice (Olsman et al., 2020). Additionally, pHIFU can disrupt and remodel the collagen and hyaluronic acid network in the ECM without causing changes in vessel size and structure (Wang et al., 2012). Several teams have investigated the use of pulsed HIFU (pHIFU) to improve the tumor targeting efficiency and the tissue penetration of nanoparticles (Lee et al., 2017; You et al., 2017). For example, You et al. (2017) injected glycol chitosan nanoparticles (FCNPs) modified with fluorescent dyes into the femoral vein of mice. The pHIFU enhanced tissue penetration and nanoparticle extravasation in mouse femoral tissue under the following treatment conditions: frequency: 1.5 MHz, duty cycle: 10%, pulse repetition frequency: 1 Hz, time: 30 s, and interval: 2 mm. However, along with the increasing treatment power, hyperthermia might be induced by the pHIFU in the target tissue, leading to histopathological abnormalities in the target tissue. Additionally, excessive pHIFU treatment might result in irreversible tissue damage and reduce the permeability of nanoparticles into tissues. Therefore, optimizing the therapeutic dose of pHIFU is particularly important in applying these pHIFU strategies for drug delivery in clinics. Hyperthermia of tissues by US action might also further dilate blood vessels and increase blood perfusion to promote the EPR effect. Fu et al. (2021) provided a therapeutic strategy to induce iron death and apoptosis in tumor cells by creating hyperthermic conditions with nanoparticles loaded with ferrate and doxorubicin combined with US. Because of the EPR effect, nanoparticles could accumulate in the tumor, and HT effectively stimulated the co-release of doxorubicin and ferrate in the tumor tissues. After 1-h US and nanoparticle treatment, tumor oxyhemoglobin saturation was enhanced from 15.4% to 36.0%. The tumor volume of mice significantly reduced after more than 15 days of treatment with nanoparticles and US.

**FIGURE 6:**
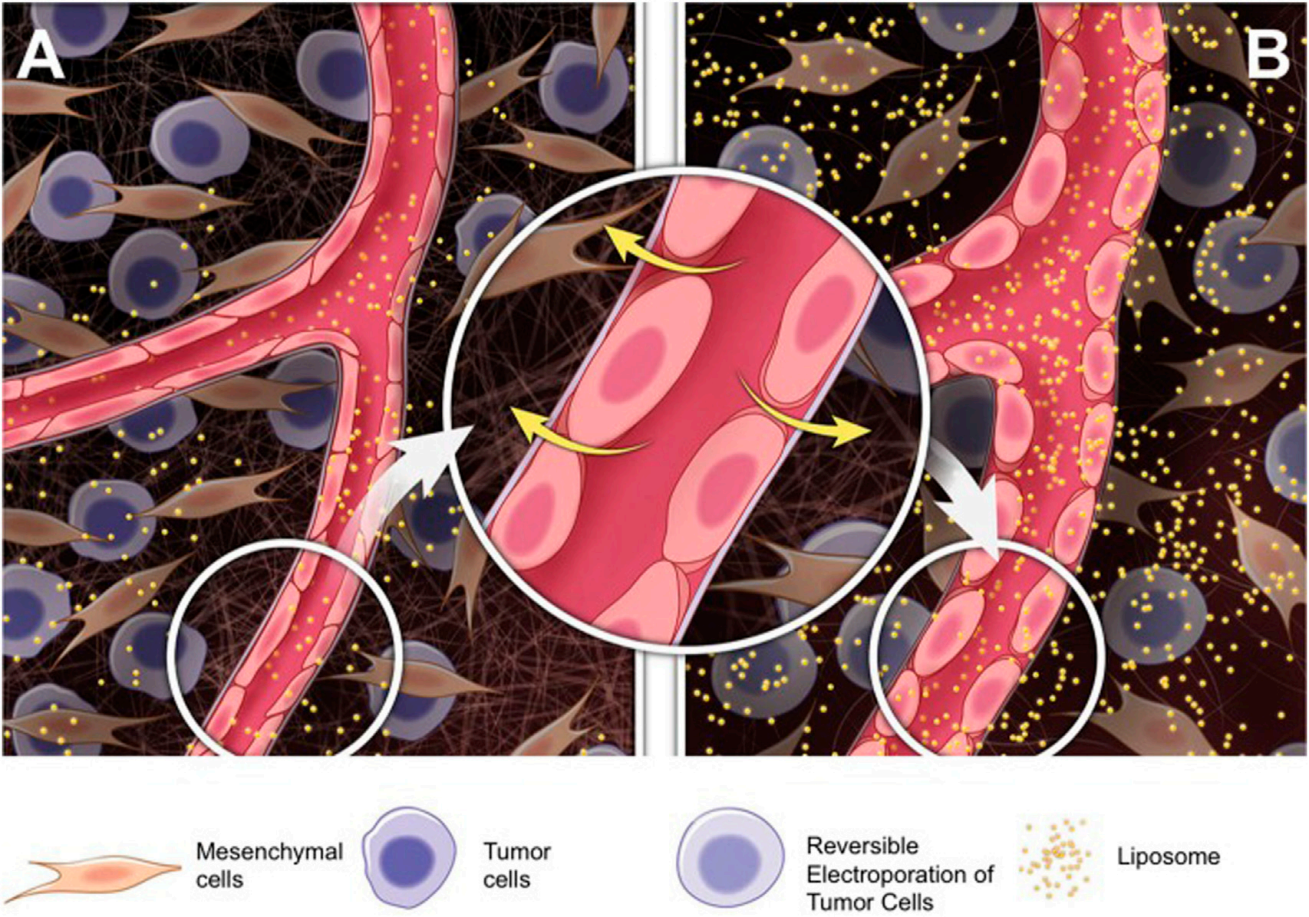
Mechanisms underlying the promotion of RE-associated vascular effects on liposomal nanoparticle delivery. Compared to **(A)** Untreated tumors, **(B)** RE induces rounding of endothelial cells and penetration of nanoparticles into treated tumors. RE, reversible electroporation (Srimathveeravalli et al., 2018). Copyright^©^ 2018, SAGE.

Electroporation (EP) has recently been studied as an “EPR effect enhancer.” Certain electric fields can cause reversible structural changes in the cell membrane, known as electroporation (Djokic et al., 2018). Besides affecting the permeability of cell membranes, electrical impulses also improve vascular permeability (Srimathveeravalli et al., 2018). Srimathveeravalli et al. (2018) studied a liposome nanoparticle (89Zr-NRep) and showed that altered vascular permeability due to EP after administration via the intravenous route might be another mode of nanoparticle entry into tumors (Figure 6). They also found that tumor absorption of nanoparticles between pre- and post-EP injections did not differ within 24 h, indicating that the vascular effect caused by EP can promote nanoparticle deposition to the tumor for some time after membrane permeability is restored. Based on the above phenomenon, they hypothesized that the vascular effect associated with reversible electroporation could improve the EPR effect and play an important role in the delivery of nanoparticles to tumors. Kodama et al. (2019) also investigated the effects of EP on tumor vasculature and the TME. They demonstrated the ability of EP to promote the aggregation of nanoparticles toward the tumor and improve the therapeutic effects. Nevertheless, the relationship between EP and EPR effects needs further investigation.

#### 3.1.2 BBB

As a microvascular network, the BBB is constructed by supporting cellular structures and brain endothelial cells, such as neurons, possibly immune cells, pericytes, and the endfeet of astrocytes. The BBB can effectively separate the central nervous system from the peripheral blood circulation (Daneman and Prat, 2015). The integrity of the BBB is critical to maintaining normal neurological function and brain homeostasis. However, most drugs used to treat brain disorders cannot cross the BBB in sufficient doses to the diseased brain tissues and cells. Therefore, there is an urgent need to develop drug delivery systems that can penetrate the BBB and deliver the drug to brain tissues. Nanoparticles are potential candidates as brain-targeted drug carriers. Nanoparticles are smaller, have lower toxicity and controlled drug-release properties, and are easily modified with proteins that target specific receptors on their surface. Nanoparticle-based DDS can cross the BBB more effectively and deliver drugs to the target site (Ahlawat et al., 2020). Nanoparticles have to be absorbed by the endothelial cells of the BBB for exocytosis to the other side with receptor-mediated endocytosis to reach the central nervous system. It has been shown that tight junctions in the endothelium of the central nervous system are opened during neuroinflammation, enhancing the transcytosis of the BBB, which allows uptake of nanoparticles (Villaseñor et al., 2019).

**FIGURE 7:**
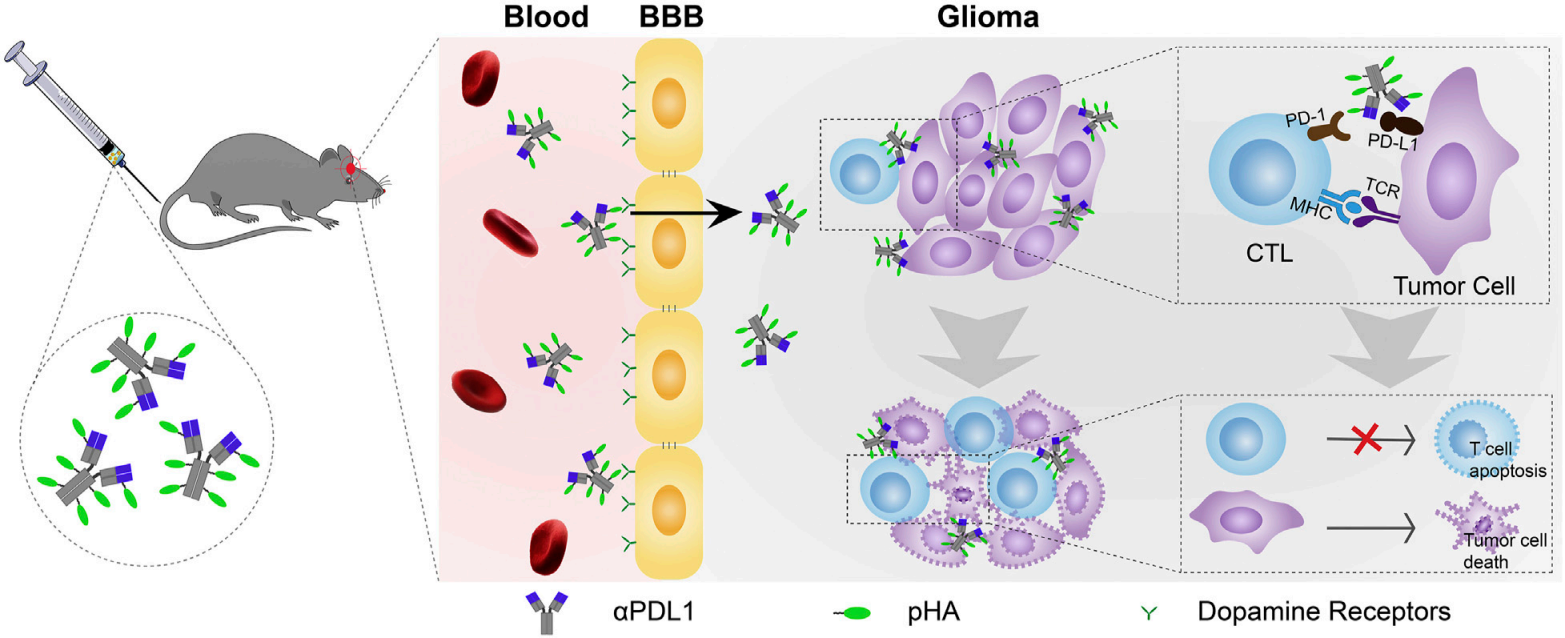
Schematic of p-hydroxybenzoic acid modification for antibody delivery crossing the BBB to treat glioma (Guo et al., 2020). Copyright^©^ 2020, Elsevier.

Nanoparticles can cross the endothelium by endocytosis and exocytosis. Transcytosis is divided into receptor-mediated transcytosis, which involves ligands, and adsorptive transcytosis, which can be used for cationic nanoparticles (Pandit et al., 2020). Nanoparticle surfaces can bind to specific ligands, such as proteins, peptides, and antibodies that specifically bind to the receptors highly expressed on the cell surface or even alone on the endothelial cells of the brain (Zhang W. et al., 2021). These ligands, often referred to as “Trojan horses,” are recognized and internalized by the transporter system on the BBB along with the associated nanoparticles to enter the brain (Choudhari et al., 2021). Commonly used receptor-ligand pairs for drug delivery to the brain include angiopep-2/LRP1, glucose/glucose transporter protein GLUT1, and transferrin/transferrin receptor (Galstyan et al., 2019). The transferrin receptor is overexpressed on brain endothelial cells and transports transferrin across the endothelium via receptor-mediated endocytosis (RMT). Transferrin/transferrin receptors have been extensively studied to increase the delivery of therapeutic agents to the brain (Johnsen et al., 2017; Fan et al., 2018). Glucose is a major energy source in the brain and is transported by the glucose transporter protein GLUT1, present in relatively high densities in BBB endothelial cells to guarantee an adequate energy supply to the brain. Fasting and the elevation of blood glucose can effectively enhance GLUT1 expression, leading to dramatically increased transportation of glucose-fixed nanoparticles across the BBB (Anraku et al., 2017). Due to the high expression in glioblastoma and BBB vascular endothelial cells, angiopep-2/LRP1 focus on glioblastoma treatment and therapy (Zhu et al., 2022). LRP1 targeted treatment can directly penetrate the BBB and deliver the drugs into brain tumor cells. Para-hydroxybenzoic acid can target dopamine receptors and promote BBB penetration via RMT. Nanoparticles modified with para-hydroxybenzoic acid could deliver PD-L1 antibody (α-PDL1), an immune checkpoint inhibitor that cannot penetrate the BBB, to the surface of glioma cells and more effectively reduce the growth of tumor cells in a glioblastoma mouse model (Figure 7) (Guo et al., 2020). The interaction of nanoparticle surface properties with the plasma membrane of endothelial cells can also be exploited for adsorption-mediated cytokinesis (AMT) into the BBB. Modifying cationic charges and conjugated compounds on nanoparticles, including heparin, cardiolipin, and lectins, can pass through the BBB (Pinheiro et al., 2021).

However, using these natural ligands, such as transferrin, glucose, insulin, leptin, or other receptor-related proteins, has limitations since they can compete with endogenous ligands, making it impossible to capture the concentration of the drug being administered (Li J. et al., 2021). The solution usually uses antibodies or peptides in place of or combination with natural ligands. For example, it is difficult to deliver sufficient drug doses to glioma patients via Angiopep-2/LRP1 administration alone due to receptor saturation effects. Moreover, combining a cell-penetrating peptide, the trans-activator of transcription (TAT) peptide, with Angiopep-2 can enhance the concentration of the drug reaching the tumor site (Zhu et al., 2022). Another study using anti-TfRA/BACE1 antibody significantly improved the BBB targeting of AuNPs without interfering with endogenous transferrin (Johnsen et al., 2018). Using physical methods to reversibly open the BBB can also facilitate the nanoparticle transport of drugs to the brain. Many physical methods have been studied, such as US, magnetic fields, and light (Tabatabaei et al., 2015; Zhang D. Y. et al., 2020; Li X. et al., 2021). Focused ultrasound (FUS) binds to intravascular microbubbles to increase BBB permeability by opening tight junctions and creating endothelial cell openings. Olsman et al. showed that combining FUS and microbubbles with transferrin receptor-targeting liposomes increases drug delivery to the brain compared to liposomes lacking BBB targeting (Olsman et al., 2021). Parameters such as pulse length and frequency should also be considered when using FUS (Lapin et al., 2020).

The transcytosis of nanoparticles by the cells is influenced by the physicochemical characteristics of the nanoparticles (e.g., size, shape and surface chemistry) as well as the experimental conditions employed (Salatin and Yari Khosroushahi, 2017). The key factors controlling BBB transport efficiency are the total ligand avidity and density on nanoparticles. Increasing the ligand density increases the affinity for the receptor, increasing the likelihood of endothelial cell internalization (Johnsen et al., 2019). However, too high densities can lead to spatial site block, reduced diffusion coefficients, and increased size of nanoparticles. Intracellular transport patterns, such as the endocytosis mediated by clathrin, also vary according to the density of the ligand (Alkilany et al., 2019). Moreover, a too-high ligand density might lead to the inability of nanoparticles to be released from the cell surface across the BBB due to high affinity (Deng et al., 2019). Therefore, the optimal ligand density is that the ligand can provide sufficient affinity for the interaction, and this interaction is compromised. For example, compared to the surface density of 10 or 50, 25% surface glucose exhibits the best BBB permeability by recognizing GLUT1 on endothelial cells (Anraku et al., 2017). Johnsen et al. also investigated the effects of TfR antibody density on the uptake and transport of nanoparticles into the brain (Johnsen et al., 2019). Safety is another issue when using these receptors for human brain-targeted delivery. For example, TfR and GLUT1 are not exclusively expressed in brain endothelial cells, implying that brain-targeting strategies might increase drug uptake in peripheral tissues (Couch et al., 2013). Therefore, targets with high expression in the BBB and low expression in other tissues must be found to improve the safety of CNS disease treatments effectively. The efficiency of TfR-targeted delivery technologies might also vary depending on the differences between species. For example, a proteomics study showed that TfR levels in mouse brain microvasculature were approximately 2.5-fold higher than in human brain microvasculature. This difference in TfR levels must be considered when we extrapolate animal data to humans (Terstappen et al., 2021).

### 3.2 Extravascular administration target binding

Besides intravenously binding to vascular targets or crossing the endothelium to reach extravascular tissues, nanoparticles can also deliver drugs through oral, inhalation, and topical administration (e.g., transdermal, intraperitoneal) without passing intravascular targets.

#### 3.2.1 Oral administration

**FIGURE 8:**
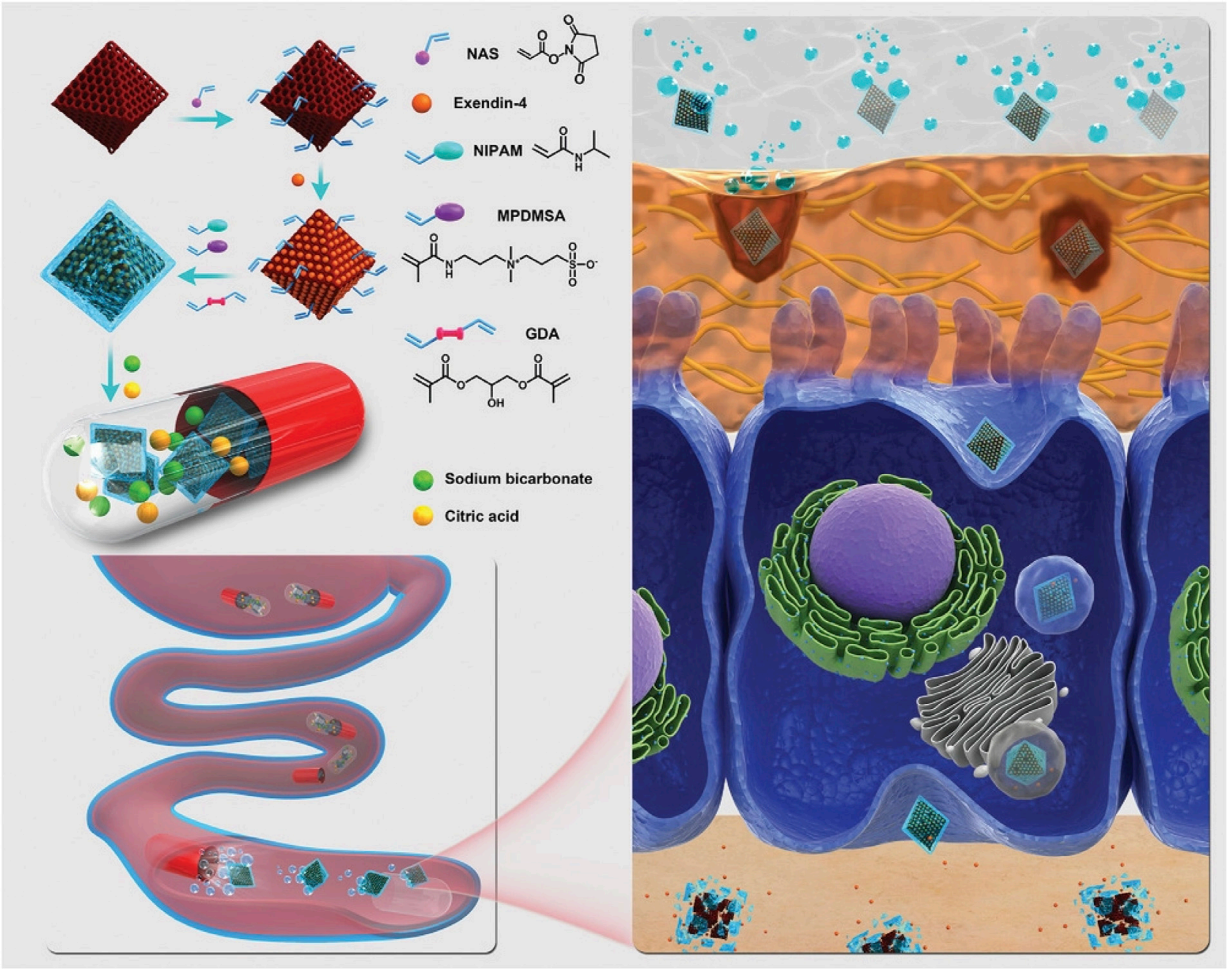
Illustration of pH-triggered self-unpacking capsule containing Ex@MIL101@Gel ± nanoparticles for oral Exendin-4 delivery. The hydrogel-coated MOF nanoparticles containing Exendin-4 were obtained by free radical copolymerization of acryloyl-modified NH2-MIL101 nanoparticles, NIPAM, and MPDMSA, with GDA as a crosslinker. The capsules protect the peptide-loaded nanoparticles from the stomach’s acidic environment and unpack them in the intestine, generating CO_2_ bubbles to promote the movement of nanoparticles. The zwitterionic surface of the nanoparticles further helps the transportation across the epithelial layer of the intestine (Zhou Y. et al., 2021). Copyright^©^ 2020, Weily.

The oral route is the most convenient, practical, and preferred administration strategy because most drugs are currently administered orally, and most patients prefer oral to intravenous administration. However, absorbing oral drugs requires overcoming some physiological barriers in the gastrointestinal tract. Nanodrug delivery platforms can transport malabsorbed drugs to the gastrointestinal tract and improve oral bioavailability. Coating nanoparticles with biological mucus modify their surface properties and increases the mucosal diffusion rate, gastrointestinal retention time, and systemic absorption (Azagury et al., 2022). Modifying ligands on the surface of nanoparticles can also specifically target the gastrointestinal tract. Azevedo et al. used genetic engineering to construct functionalized recombinant albumin with targeted modification on the surface of PLGA-PEG nanoparticles to improve nanoparticle transport across the intestinal epithelium by enhancing the binding between nanoparticles and intestinal neonatal Fc receptors (FcRn) (Azevedo et al., 2020). The delivery of peptides/proteins readily degraded by the gastrointestinal tract via nanoparticles provides an alternative management measure for diabetic patients. Furthermore, Zhou et al. designed a PH-triggered release of Exendin-4 capsules (Figure 8). Exendin-4, the active ingredient in the treatment of diabetes, was efficiently loaded and immobilized on a metal-organic framework (MOF). The amphoteric hydrogel layer coated on the skeleton surface has a unique ability to permeate across the cellular mucus layer and allows for effective internalization of the nanocarrier by epithelial cells. To protect the nanoparticles from destruction in the stomach, a pH-sensitive capsule is used to carry the drug, which is insoluble in an acidic environment and dissolves rapidly in the intestinal fluid, a neutral environment. Based on the fluorescence analysis, MOF nanoparticles led to enhanced absorption of Exendin-4 in the intestine compared to free Exendin-4. After oral administration, the nanoparticles significantly increased the plasma Exendin-4 level and promoted endogenous insulin secretion in diabetic rats, which had significant hypoglycemic effects (Zhou Y. et al., 2021). The use of nanoparticles for insulin transport or gene delivery has also shown excellent therapeutic effects in diabetic model mice (Lamson et al., 2020; Sudhakar et al., 2020). Besides improving diabetes treatment options, oral nanoparticles have shown great therapeutic promise in other diseases, such as intestinal disorders, bacterial infectious diseases, and oncology (Kim et al., 2019; Shen et al., 2019; Ndayishimiye et al., 2021; Wang J. et al., 2021; Yu et al., 2021; Li et al., 2022; Zhang G. et al., 2022). Additionally, a pioneering attempt to synthesize orally administered nanoparticles has recently been conducted by researchers to easily prepare and use gold nanoparticles (GNP) (Ndayishimiye et al., 2021) in the synthesis conditions. Wang et al. demonstrated that antibacterial GNP (A-GNP) could be synthesized *in vivo* by oral administration of two raw materials: aminophenyl boronic acid (ABA) and tetrachloroauric acid (HAuCl_4_). A-GNP has a good antibacterial effect, long half-life (16–17 h), high effective clearance (residual concentration close to 0 *in vivo* within 72 h), and high biosafety (Wang L. et al., 2021).

Notably, the size and shape of nanoparticles show importance in utilizing endocytosis through the gastrointestinal tract and subsequent exocytosis. For example, the large surface area of the polymer NPs improves the efficiency of drug absorption by increasing the number of interactions with the gastrointestinal tract (Banerjee et al., 2016). The uptake of rod-shaped nanoparticles by epithelial cells is usually superior to that of spherical particles (Banerjee et al., 2016). Pathologies such as inflammation may increase the permeability of the intestinal epithelium and alter mucus production, pH and gastrointestinal microbiota, thus affecting the delivery of nanomedicines (Durán-Lobato et al., 2020).

#### 3.2.2 Inhalation

Pulmonary inhalation is an attractive route of drug delivery in addition to oral and injectable administrations. Therapeutic drugs can be delivered by inhalation into the pulmonary airways to treat diseases such as cystic fibrosis (CF), chronic obstructive pulmonary disease (COPD) and asthma, and lung cancer (Edwards et al., 1998; Chow et al., 2007). Physiological and anatomical characteristics of the lungs, such as large absorption area, high permeability of the alveolar epithelial membrane, and high blood flow, make inhalation drug delivery an attractive non-invasive route of administration that avoids drug degradation in the gastrointestinal tract, reduces side effects on normal tissues and organs, and avoids first-pass metabolism in the liver (Kuzmov and Minko, 2015). However, most free drugs, nucleic acids, and peptides used for therapeutic purposes cannot be delivered to the lungs naturally by inhalation. Hence, there has been an increasing interest in delivering drugs to the lungs by inhalation with nanotechnology-based delivery systems.

Although inhaled nanoparticles offer a unique opportunity to treat pulmonary airway diseases, airway physiology and pulmonary clearance mechanisms are key barriers to effective nanoparticle deposition and preservation in the lung (Mangal et al., 2017). The airway surface layer (ASL) is the first line of defense of the respiratory system, protecting the lungs’ airways from invasion by harmful foreign substances such as pathogens and pollutants. The ASL consists of the luminal mucus gel layer that covers the airway epithelium and the periciliary layer (PLC). Aspirated foreign bodies, including therapeutic nanoparticles, are readily captured by the mucus gel layer and rapidly cleared from the lungs by mucociliary clearance (MCC), continuous cilia beating of the PCL, or coughing (Chen D. et al., 2021). The mucin glycoprotein in the mucus gel layer has negatively charged O-glycosylated domains. Due to ionic interactions, positively charged nanoparticles cannot pass through the mucus layer (Zhong et al., 2016). The barrier properties of the mucus gel layer are usually more pronounced in disease states. For example, the mucin concentration in the airway mucus of CF and COPD patients is usually elevated, which tightens the mucus mesh spacing and makes it more difficult for nanoparticles to penetrate the airway mucus (Duncan et al., 2016; Chisholm et al., 2019).

Coating nanoparticle surfaces with hydrophilic and neutrally charged PEG polymers to provide an inert mucosal surface can effectively cross the mucus barrier and reduce alveolar macrophage uptake through chemical binding or physical adsorption methods (Huckaby and Lai, 2018). The nanoparticles modified by PEG in the airway mucus of CF and COPD mice exhibited considerable permeability and were able to be uniformly distributed in the airway, and their retention time was significantly prolonged (Schneider et al., 2017; De Leo et al., 2018; Osman et al., 2018). Recently, PEGylated nanoparticles with efficient transmucosal delivery capacity significantly reduced the development of fibrosis and improved lung function in a mouse model of bleomycin-induced pulmonary fibrosis (Bai et al., 2022). Several PEG alternatives have also been developed to improve the penetration of particles through the airway mucus. Ge et al. used fluorinated polymers containing cationic polypeptides that have a unique ability to resist mucosal adhesion and maintain physiological stability due to fluorination, significantly enhancing the ability of nanoparticles to penetrate mucus. They showed that siRNAs targeting tumor necrosis factor (TNF)-α delivered by fluorinated nanoparticles reduced TNF-α expression and pro-inflammatory responses to a greater extent in the lungs of mice of acute lung injury compared to non-fluorinated formulations that do not penetrate airway mucus (Ge et al., 2020). Self-emulsifying drug delivery systems have been developed as gene-delivery vehicles that can effectively improve the passage through CF airway mucus (Griesser et al., 2019). Recent studies have also shown that ligand modification strategies of nanoparticles using folic acid, transferrin, and neonatal Fc receptor ligand (FcBP) can enhance the lung retention effect of nanoparticles (Rosière et al., 2018; Parvathaneni et al., 2021; Yu et al., 2023). For example, Parvathaneni et al. used transferrin ligand-modified nanoparticles (Tf-AMQ NPs) to treat non-small cell lung cancer, which effectively penetrated the tumor core and inhibited tumor growth. In A549 and H1299 cells, Tf-AMQ NPs significantly induced apoptosis and reduced colony growth % (Parvathaneni et al., 2021).

The physicochemical properties of nanoparticles, such as particle size, can affect their lung retention efficiency (Shen and Minko, 2020). Furthermore, a team has investigated the effect of nanoparticle hardness on pulmonary drug delivery and showed that hard nanoparticles have better endocytosis than soft nanoparticles, which might be related to actin filaments- and Ca^2+^-mediated endocytosis (Yu et al., 2023). The cytotoxicity of nanomaterials should be considered along with its mucus penetration and lung retention efficiency. Clinical and experimental data suggest that the size and shape of nanoparticles affect their toxicity to the lungs, with smaller nanoparticle sizes and larger surface areas causing more damage to the lungs (Nel et al., 2006; Osman et al., 2020). Besides the physicochemical properties of nanoparticles, alveolar sizes of different sexes, health conditions, and different species might also affect drug inhalation and deposition (Xi et al., 2020). At the same time, for inhalation drug delivery, specific devices for nebulization and inhalation are usually applied. Therefore, how to maintain the specific physicochemical properties of nanoparticles during nebulization without aggregation is an issue to consider during design and preparation (Yhee et al., 2016).

#### 3.2.3 Topical administration

Besides oral and inhalation drug delivery, nano-DDS can also be used for topical administration. In traditional topical drug delivery methods, the residence time and the duration of the drug effect in the tissue are short. Frequent drug administration may increase the risk of local pain and infection (Zhu et al., 2020). Intra-articular administration can significantly increase local drug concentration, prolong the drug’s half-life and reduce the adverse effects of systemic drug delivery (Bedingfield et al., 2021; Mao et al., 2021; Wei et al., 2021). He et al. designed a DDS using pH-sensitive polyacrylic acid (PAA)-modified mesoporous silica nanoparticles (MSNs) loaded with andrographolide (AG) to form the AG@MSNs-PAA nanoplatform. The nanoparticles had uniform size (120 nm), high drug loading efficiency [(22.38 ± 0.71)%], and pH-responsive properties (as shown), facilitating their sustained release in osteoarthritis (OA). Moreover, the AG@MSNs-PAA exhibited stronger anti-arthritic efficacy and cartilage protection than AG in cellular assays and OA rat models (He et al., 2021). Intra-articular injection of high-molecular-weight (HMW) HAs is one of the current treatment options for OA. However, the HMW HA treatment can relieve patients’ pain but has side effects, such as local inflammation. The mechanism triggering inflammation might be the degradation of HMW HAs into LMW HA molecules by hyaluronidase, associated with increased catabolic gene expression and pro-inflammatory cytokine production (Knudson et al., 2019). Kang et al. (2021) reported a self-assembled hyaluronic acid nanoparticle (HA-NP) as a potential therapeutic agent for OA treatment. HA-NP showed *in vitro* resistance to digestion with hyaluronidase and *in vivo* long-term retention ability in knee joint, compared to free HMW HA. They showed that HA-NP could treat OA by interfering with fragmented LMW HA-CD44 interactions and the underlying mechanisms involved in OA pathogenesis and progression.

One type of non-adhesive nanoparticles (NNPs) consisting of block copolymer poly (lactic acid)-hyperbranched poly (glycerol) (PLA-HPG) can be converted into bioadhesive nanoparticles (BNPs) by brief incubation with sodium periodate (Deng et al., 2016). This treatment alters the chemistry of the HPG molecule, converting vicinal diols into aldehydes, giving them the covalently bonded molecular adhesion that results from the interaction of surface aldehydes with amino groups on biomolecules, such as stratum corneum, the extracellular space, cell surfaces, and the tumor’s protein-rich matrix (Thavarajah et al., 2012). BNPs can be used as a local carrier of chemotherapeutic drugs, through intraperitoneal delivery to peritoneal carcinoma, intracranial delivery to brain tumors, and intratumoral delivery to squamous cell carcinoma of the skin, through the skin for psoriasis (Deng et al., 2016; Song et al., 2017; Hu et al., 2021; Mai et al., 2022). They are also used intravaginally to increase the retention of antiviral drugs in the reproductive tract (Mohideen et al., 2017). Nanomaterials have gained attention as drug carriers for transdermal drug delivery. The skin acts as a natural barrier that prevents most drugs from penetrating the highly ordered and dense stratum corneum, limiting the use of transdermal drug delivery. Some lipid nano vehicles and polymeric nanoparticles can overcome the barrier function of the stratum corneum and improve the stability and delivery efficiency of drugs (Lapteva et al., 2014; Sala et al., 2018). Zhao et al. (2022) prepared a highly dispersed DES-MSNs system by modifying citric acid and amino acid on the surface of Mesoporous silica nanoparticles (MSNs), which enables the nanoparticles across SC to the deeper skin layers. This work provides a new strategy for the controlled and sustained delivery of nanoparticles. A recent study found that intraperitoneal injection of anionic nanoparticles can selectively accumulate in TAMs. In a mouse model of metastatic ovarian cancer, fluorescently labeled silica poly (lactic acid-ethanolic acid) and polystyrene nanoparticles selectively accumulated in TAMs. Quantitative absorption of silica particles indicated that more than 80% of the injected dose accumulated in TAMs. This targeting result was mediated by active, selective uptake by TAMs, and the targeting properties and efficiency are difficult to achieve by intravenous drug delivery (Haber et al., 2020). Other researchers have also infused CAT-TCPP/FCS nanoparticles into the bladder cavity via intravesical perfusion. The results showed that CAT-TCPP/FCS NPs have good transmucosal and intratumoral penetration ability and can catalyze O_2_ generation from tumor endogenous H_2_O_2_ via peroxidase, which can effectively relieve tumor tissue hypoxia and improve bladder efficacy *in situ* tumor under SDT ablation ultrasound (Li G. et al., 2020). Interestingly, it is also possible to spray nanomedicines directly on the surface of the heart. The authors synthesized P/B-COS nanoparticles by loading the glucocorticoid anti-inflammatory drug budesonide (BUD) into PLGA and chitosan (COS) to form a stable saline solution. The anti-inflammatory effect was produced by spraying nanoparticles directly into the local myocardium by catheter saline infusion during atrial fibrillation ablation procedures. Due to the effects of COS, the nanoparticles are positively charged and can better adhere to negatively charged cell membranes, improving the efficiency of local drug delivery (Liu et al., 2022). Topical nanoparticle drug delivery can address the inefficiency of hydrophilic or macromolecular drug delivery and the difficulty to overcome physiological barriers, but these studies are still at the research stage. The translation of such drug delivery is equally limited by the variability between animal models and humans. Although nanoparticle systems have been successfully developed at the laboratory scale, the challenges of scaling up and the lack of reproducibility have made it difficult to achieve conversion from laboratory scale to industrial scale (Gupta et al., 2017).

## 4 Limitations and challenges of nano-targeted delivery systems

For the specific nature of nanoparticles, nanoparticles researcher has been produced promising results in animal models and *in vitro* studies. However, most research is only at the research stage, the benefits to patients are not nearly enough. The construction of a targeted nanomedicine delivery system is complex task and requires multiple targeting designs that take into account the physiological variables such as blood flow, disease status and tissue structure (Patra et al., 2018). The variability between animal models and humans, the function of nanodrugs in humans is something that is currently difficult for researchers to grasp. Also due to inter-patient heterogeneity, this limitation may make targeted nanoparticles patient-specific in their distribution and function and prevent their widespread use. Notably, the growing popularity of precision or personalized medicine may enable targeted drug delivery systems to play an important role (Collins and Varmus, 2015). The advent of precision medicine minimizes the impact of patient heterogeneity, and the development of individualized treatment plans for each patient can enable nanomedicines to achieve their optimal efficacy. When surface modification of targeted nanoparticles is engineered, the ratio of receptors to ligands needs to be considered so that they can bind adequately to the cell surface. In order to achieve specific targeted drug delivery, the target should be selected in a way that avoids or reduces the expression of the target on healthy cells. The binding rate between the nanoparticles and the modified molecules should also be considered to avoid side effects caused by unsuitable binding rates (Hoshyar et al., 2016; Kou et al., 2018).

Many studies have attributed the accumulation of nanoparticles in tumors to the EPR effect. However, there are also reports that contradict this conclusion. A recent study utilizing imaging techniques in a mouse tumor model has determined that only a small fraction of nanoparticles accumulation in tumors can be attributed to passive transport, including the EPR effect. Other mechanisms such as immune cell interactions, protein crowns and molecular mechanisms may promote nanoparticles accumulation (Sindhwani et al., 2020). Therefore, while continuing to explore the use of the EPR effect to promote nanoparticle accumulation, the delivery and distribution of nanoparticles should be quantitatively evaluated. Meanwhile tumors and the biological barriers surrounding them may be far more complex to study. Mucus throughout the body, especially in the gastrointestinal tract and lungs, plays a huge role in hindering the delivery of drugs by targeted nanoparticles. In particular, the patient’s lifestyle, disease state and other factors can lead to different properties of the mucus, making the barrier faced by the nanoparticles more complex (Wan et al., 2020).

Hybrid nanocarriers are currently one of the most promising tools, as they have different properties in a single system, thus ensuring enhanced performance of the material in the therapeutic system. Nevertheless, the mechanisms of action and toxicity of drug delivery systems are still poorly understood, which provides opportunities for new research. There are still high uncertainties in the clinical application of targeted nanoparticles, such as assessing the safety and toxicity of nanomaterials, the lack of effective regulation, and the stability of nanomedicines when they are introduced into biological systems (Patra et al., 2018).

## 5 Summary and future outlook

We summarized and discussed the recent application of nanomaterial-based delivery systems in intra- and extravascular diseases. Through different modifications of nanomaterials, such as active targeting, bionanotechnology strategies, and combining exogenous physical triggering strategies, nanomaterials can improve the shortcomings of traditional drugs that are easily cleared in the body with a short half-life and undesirable side effects on vital organs, as well as small molecule drugs that cannot enter the BBB for targeted delivery. Therefore, drug delivery efficiency can improve in vascular diseases, tumors, and neurological diseases by different drug delivery methods. Although the application of nanomaterials has achieved promising therapeutic effects in these diseases, we should pay attention to the heterogeneity of animal models and human diseases and the heterogeneity between different development periods of the same disease and different patients. We should carefully think before applying animal data to human diseases. To better address this heterogeneity, both treatments and delivery systems can be personalized for specific patients. Future experimental research should consider whether the therapeutic purpose can be achieved and focus on the synthesis and design of nanomaterials. For nanomaterials with relatively simple structures, a clinical translation should also be considered to provide a basis for future nanotechnology development and clinical translation in various clinical diseases. The use of nano-drug delivery systems to deliver precise amount of drug to target cells without interfering with the physiological functions of healthy cells is a trend and goal in the field of research and development for the coming decades. With the development of nanotechnology and delivery strategies, a deeper development of nano DDS is expected for various diseases in clinical practice.

## References

[B1] Abumanhal-Masarweh H. Koren L. Zinger A. Yaari Z. Krinsky N. Kaneti G. (2019). Sodium bicarbonate nanoparticles modulate the tumor pH and enhance the cellular uptake of doxorubicin. J. Control Release 296, 1–13. 10.1016/j.jconrel.2019.01.004 30615983 PMC6660974

[B2] Ahlawat J. Guillama Barroso G. Masoudi Asil S. Alvarado M. Armendariz I. Bernal J. (2020). Nanocarriers as potential drug delivery candidates for overcoming the blood-brain barrier: Challenges and possibilities. ACS Omega 5 (22), 12583–12595. 10.1021/acsomega.0c01592 32548442 PMC7288355

[B3] Aird W. C. (2008). Endothelium in health and disease. Pharmacol. Rep. 60 (1), 139–143. 18276995

[B4] Aizik G. Waiskopf N. Agbaria M. Ben-David-Naim M. Nordling-David M. M. Jbara-Agbaria D. (2020). Targeting and imaging of monocyte-derived macrophages in rat's injured artery following local delivery of liposomal quantum dots. J. Control Release 318, 145–157. 10.1016/j.jconrel.2019.12.009 31830540

[B5] Alapan Y. Yasa O. Schauer O. Giltinan J. Tabak A. F. Sourjik V. (2018). Soft erythrocyte-based bacterial microswimmers for cargo delivery. Sci. Robot. 3 (17), eaar4423. 10.1126/scirobotics.aar4423 33141741

[B6] Alkilany A. M. Zhu L. Weller H. Mews A. Parak W. J. Barz M. (2019). Ligand density on nanoparticles: A parameter with critical impact on nanomedicine. Adv. Drug Deliv. Rev. 143, 22–36. 10.1016/j.addr.2019.05.010 31158406

[B7] Amengual J. Barrett T. J. (2019). Monocytes and macrophages in atherogenesis. Curr. Opin. Lipidol. 30 (5), 401–408. 10.1097/mol.0000000000000634 31361625 PMC7809604

[B8] Anraku Y. Kuwahara H. Fukusato Y. Mizoguchi A. Ishii T. Nitta K. (2017). Glycaemic control boosts glucosylated nanocarrier crossing the BBB into the brain. Nat. Commun. 8 (1), 1001. 10.1038/s41467-017-00952-3 29042554 PMC5645389

[B9] Arias S. L. Shetty A. Devorkin J. Allain J. P. (2018). Magnetic targeting of smooth muscle cells *in vitro* using a magnetic bacterial cellulose to improve cell retention in tissue-engineering vascular grafts. Acta Biomater. 77, 172–181. 10.1016/j.actbio.2018.07.013 30004023

[B10] Azagury A. Baptista C. Milovanovic K. Shin H. Morello P. 3rd Perez-Rogers J. (2022). Biocoating-A critical step governing the oral delivery of polymeric nanoparticles. Small 18 (26), e2107559. 10.1002/smll.202107559 35606684 PMC9250634

[B11] Azevedo C. Nilsen J. Grevys A. Nunes R. Andersen J. T. Sarmento B. (2020). Engineered albumin-functionalized nanoparticles for improved FcRn binding enhance oral delivery of insulin. J. Control Release 327, 161–173. 10.1016/j.jconrel.2020.08.005 32771477

[B12] Bai X. Zhao G. Chen Q. Li Z. Gao M. Ho W. (2022). Inhaled siRNA nanoparticles targeting IL11 inhibit lung fibrosis and improve pulmonary function post-bleomycin challenge. Sci. Adv. 8 (25), eabn7162. 10.1126/sciadv.abn7162 35731866 PMC9216512

[B13] Banerjee A. Qi J. Gogoi R. Wong J. Mitragotri S. (2016). Role of nanoparticle size, shape and surface chemistry in oral drug delivery. J. Control Release 238, 176–185. 10.1016/j.jconrel.2016.07.051 27480450 PMC5289391

[B14] Barenholz Y. (2012). Doxil®-the first FDA-approved nano-drug: Lessons learned. J. Control Release 160 (2), 117–134. 10.1016/j.jconrel.2012.03.020 22484195

[B15] Bedingfield S. K. Colazo J. M. Yu F. Liu D. D. Jackson M. A. Himmel L. E. (2021). Amelioration of post-traumatic osteoarthritis via nanoparticle depots delivering small interfering RNA to damaged cartilage. Nat. Biomed. Eng. 5 (9), 1069–1083. 10.1038/s41551-021-00780-3 34413494 PMC8497446

[B16] Bellini M. Riva B. Tinelli V. Rizzuto M. A. Salvioni L. Colombo M. (2020). Engineered ferritin nanoparticles for the bioluminescence tracking of nanodrug delivery in cancer. Small 16 (39), e2001450. 10.1002/smll.202001450 32856404

[B17] Ben-Akiva E. Meyer R. A. Yu H. Smith J. T. Pardoll D. M. Green J. J. (2020). Biomimetic anisotropic polymeric nanoparticles coated with red blood cell membranes for enhanced circulation and toxin removal. Sci. Adv. 6 (16), eaay9035. 10.1126/sciadv.aay9035 32490199 PMC7239698

[B18] Boada C. Zinger A. Tsao C. Zhao P. Martinez J. O. Hartman K. (2020). Rapamycin-loaded biomimetic nanoparticles reverse vascular inflammation. Circ. Res. 126 (1), 25–37. 10.1161/circresaha.119.315185 31647755

[B19] Boateng F. Ngwa W. (2019). Delivery of nanoparticle-based radiosensitizers for radiotherapy applications. Int. J. Mol. Sci. 21 (1), 273. 10.3390/ijms21010273 31906108 PMC6981554

[B20] Borsig L. Wong R. Feramisco J. Nadeau D. R. Varki N. M. Varki A. (2001). Heparin and cancer revisited: Mechanistic connections involving platelets, P-selectin, carcinoma mucins, and tumor metastasis. Proc. Natl. Acad. Sci. U. S. A. 98 (6), 3352–3357. 10.1073/pnas.061615598 11248082 PMC30657

[B21] Bourquin J. Milosevic A. Hauser D. Lehner R. Blank F. Petri-Fink A. (2018). Biodistribution, clearance, and long-term fate of clinically relevant nanomaterials. Adv. Mater 30 (19), e1704307. 10.1002/adma.201704307 29389049

[B22] Charest G. Tippayamontri T. Shi M. Wehbe M. Anantha M. Bally M. (2020). Concomitant chemoradiation therapy with gold nanoparticles and platinum drugs Co-encapsulated in liposomes. Int. J. Mol. Sci. 21 (14), 4848. 10.3390/ijms21144848 32659905 PMC7402338

[B23] Chen M. Chen M. He J. (2019). Cancer cell membrane cloaking nanoparticles for targeted co-delivery of doxorubicin and PD-L1 siRNA. Artif. Cells Nanomed Biotechnol. 47 (1), 1635–1641. 10.1080/21691401.2019.1608219 31027450

[B24] Chen D. Liu J. Wu J. Suk J. S. (2021). Enhancing nanoparticle penetration through airway mucus to improve drug delivery efficacy in the lung. Expert Opin. Drug Deliv. 18 (5), 595–606. 10.1080/17425247.2021.1854222 33218265 PMC9479118

[B25] Chen Y. Li Z. H. Pan P. Zeng R. Y. Zhang X. Z. (2021). Tumor-specific ONOO(-) nanogenerator for improved drug delivery and enhanced chemotherapy of tumor. ACS Nano 15 (7), 11514–11525. 10.1021/acsnano.1c01312 34275285

[B26] Chen B. Liu X. Li Y. Shan T. Bai L. Li C. (2022). iRGD tumor-penetrating peptide-modified nano-delivery system based on a marine sulfated polysaccharide for enhanced anti-tumor efficiency against breast cancer. Int. J. Nanomed. 17, 617–633. 10.2147/ijn.S343902 PMC8842734 35173433

[B27] Chen L. Zhou Z. Hu C. Maitz M. F. Yang L. Luo R. (2022). Platelet membrane-coated nanocarriers targeting plaques to deliver anti-CD47 antibody for atherosclerotic therapy. Res. (Wash D C) 2022, 9845459. 10.34133/2022/9845459 PMC8791388 35118420

[B28] Cheng Y. Ren J. Fan S. Wu P. Cong W. Lin Y. (2022). Nanoparticulates reduce tumor cell migration through affinity interactions with extracellular migrasomes and retraction fibers. Nanoscale Horiz. 7 (7), 779–789. 10.1039/d2nh00067a 35703339

[B29] Chisholm J. F. Shenoy S. K. Shade J. K. Kim V. Putcha N. Carson K. A. (2019). Nanoparticle diffusion in spontaneously expectorated sputum as a biophysical tool to probe disease severity in COPD. Eur. Respir. J. 54 (2), 1900088. 10.1183/13993003.00088-2019 31164433 PMC8081045

[B30] Choi K. Y. Han H. S. Lee E. S. Shin J. M. Almquist B. D. Lee D. S. (2019). Hyaluronic acid-based activatable nanomaterials for stimuli-responsive imaging and therapeutics: Beyond CD44-mediated drug delivery. Adv. Mater 31 (34), e1803549. 10.1002/adma.201803549 30773699

[B31] Choudhari M. Hejmady S. Narayan Saha R. Damle S. Singhvi G. Alexander A. (2021). Evolving new-age strategies to transport therapeutics across the blood-brain-barrier. Int. J. Pharm. 599, 120351. 10.1016/j.ijpharm.2021.120351 33545286

[B32] Chow A. H. Tong H. H. Chattopadhyay P. Shekunov B. Y. (2007). Particle engineering for pulmonary drug delivery. Pharm. Res. 24 (3), 411–437. 10.1007/s11095-006-9174-3 17245651

[B33] Chu D. Dong X. Shi X. Zhang C. Wang Z. (2018). Neutrophil-based drug delivery systems. Adv. Mater 30 (22), e1706245. 10.1002/adma.201706245 29577477 PMC6161715

[B34] Clausen A. S. Østergaard D. E. Holmberg P. Henriksen J. R. Tham J. Damborg P. P. (2020). Quantitative determination of (64)Cu-liposome accumulation at inflammatory and infectious sites: Potential for future theranostic system. J. Control Release 327, 737–746. 10.1016/j.jconrel.2020.09.018 32920081

[B35] Collins F. S. Varmus H. (2015). A new initiative on precision medicine. N. Engl. J. Med. 372 (9), 793–795. 10.1056/NEJMp1500523 25635347 PMC5101938

[B36] Couch J. A. Yu Y. J. Zhang Y. Tarrant J. M. Fuji R. N. Meilandt W. J. (2013). Addressing safety liabilities of TfR bispecific antibodies that cross the blood-brain barrier. Sci. Transl. Med. 5 (183), 183ra57, 1–12. 10.1126/scitranslmed.3005338 23636093

[B37] Daneman R. Prat A. (2015). The blood-brain barrier. Cold Spring Harb. Perspect. Biol. 7 (1), a020412. 10.1101/cshperspect.a020412 25561720 PMC4292164

[B38] Davies M. J. Gordon J. L. Gearing A. J. Pigott R. Woolf N. Katz D. (1993). The expression of the adhesion molecules ICAM-1, VCAM-1, PECAM, and E-selectin in human atherosclerosis. J. Pathol. 171 (3), 223–229. 10.1002/path.1711710311 7506307

[B39] Davies P. F. Civelek M. Fang Y. Fleming I. (2013). The atherosusceptible endothelium: Endothelial phenotypes in complex haemodynamic shear stress regions *in vivo* . Cardiovasc Res. 99 (2), 315–327. 10.1093/cvr/cvt101 23619421 PMC3695748

[B40] Davis M. E. Chen Z. G. Shin D. M. (2008). Nanoparticle therapeutics: An emerging treatment modality for cancer. Nat. Rev. Drug Discov. 7 (9), 771–782. 10.1038/nrd2614 18758474

[B41] de Jonge M. J. Verweij J. (2006). Renal toxicities of chemotherapy. Semin. Oncol. 33 (1), 68–73. 10.1053/j.seminoncol.2005.11.011 16473645

[B42] De Leo V. Ruscigno S. Trapani A. Di Gioia S. Milano F. Mandracchia D. (2018). Preparation of drug-loaded small unilamellar liposomes and evaluation of their potential for the treatment of chronic respiratory diseases. Int. J. Pharm. 545 (1-2), 378–388. 10.1016/j.ijpharm.2018.04.030 29678545

[B43] de Saint Victor M. Barnsley L. C. Carugo D. Owen J. Coussios C. C. Stride E. (2019). Sonothrombolysis with magnetically targeted microbubbles. Ultrasound Med. Biol. 45 (5), 1151–1163. 10.1016/j.ultrasmedbio.2018.12.014 30773375

[B44] Deng Y. Yang F. Cocco E. Song E. Zhang J. Cui J. (2016). Improved i.p. drug delivery with bioadhesive nanoparticles. Proc. Natl. Acad. Sci. U. S. A. 113 (41), 11453–11458. 10.1073/pnas.1523141113 27663731 PMC5068292

[B45] Deng H. Dutta P. Liu J. (2019). Stochastic modeling of nanoparticle internalization and expulsion through receptor-mediated transcytosis. Nanoscale 11 (23), 11227–11235. 10.1039/c9nr02710f 31157808 PMC6634982

[B46] Dhaliwal A. Zheng G. (2019). Improving accessibility of EPR-insensitive tumor phenotypes using EPR-adaptive strategies: Designing a new perspective in nanomedicine delivery. Theranostics 9 (26), 8091–8108. 10.7150/thno.37204 31754383 PMC6857058

[B47] Ding Z. Wang D. Shi W. Yang X. Duan S. Mo F. (2020). *In vivo* targeting of liver cancer with tissue- and nuclei-specific mesoporous silica nanoparticle-based nanocarriers in mice. Int. J. Nanomed. 15, 8383–8400. 10.2147/ijn.S272495 PMC7605659 33149582

[B48] Djokic M. Cemazar M. Popovic P. Kos B. Dezman R. Bosnjak M. (2018). Electrochemotherapy as treatment option for hepatocellular carcinoma, a prospective pilot study. Eur. J. Surg. Oncol. 44 (5), 651–657. 10.1016/j.ejso.2018.01.090 29402556

[B49] Doherty G. J. Tempero M. Corrie P. G. (2018). HALO-109-301: A phase III trial of PEGPH20 (with gemcitabine and nab-paclitaxel) in hyaluronic acid-high stage IV pancreatic cancer. Future Oncol. 14 (1), 13–22. 10.2217/fon-2017-0338 29235360

[B50] Dong X. Chu D. Wang Z. (2017). Leukocyte-mediated delivery of nanotherapeutics in inflammatory and tumor sites. Theranostics 7 (3), 751–763. 10.7150/thno.18069 28255364 PMC5327647

[B51] Dosta P. Tamargo I. Ramos V. Kumar S. Kang D. W. Borrós S. (2021). Delivery of anti-microRNA-712 to inflamed endothelial cells using poly(β-amino ester) nanoparticles conjugated with VCAM-1 targeting peptide. Adv. Healthc. Mater 10 (15), e2001894. 10.1002/adhm.202001894 33448151 PMC8277885

[B52] Duan L. Yang L. Jin J. Yang F. Liu D. Hu K. (2020). Micro/nano-bubble-assisted ultrasound to enhance the EPR effect and potential theranostic applications. Theranostics 10 (2), 462–483. 10.7150/thno.37593 31903132 PMC6929974

[B53] Duncan G. A. Jung J. Joseph A. Thaxton A. L. West N. E. Boyle M. P. (2016). Microstructural alterations of sputum in cystic fibrosis lung disease. JCI Insight 1 (18), e88198. 10.1172/jci.insight.88198 27812540 PMC5085601

[B54] Dunne M. Regenold M. Allen C. (2020). Hyperthermia can alter tumor physiology and improve chemo- and radio-therapy efficacy. Adv. Drug Deliv. Rev. 163-164, 98–124. 10.1016/j.addr.2020.07.007 32681862

[B55] Durán-Lobato M. Niu Z. Alonso M. J. (2020). Oral delivery of biologics for precision medicine. Adv. Mater 32 (13), e1901935. 10.1002/adma.201901935 31222910

[B56] Edwards D. A. Ben-Jebria A. Langer R. (1998). Recent advances in pulmonary drug delivery using large, porous inhaled particles. J. Appl. Physiol. 85 (2), 379–385. 10.1152/jappl.1998.85.2.379 9688708

[B57] Fan K. Jia X. Zhou M. Wang K. Conde J. He J. (2018). Ferritin nanocarrier traverses the blood brain barrier and kills glioma. ACS Nano 12 (5), 4105–4115. 10.1021/acsnano.7b06969 29608290

[B58] Fang R. H. Hu C. M. Luk B. T. Gao W. Copp J. A. Tai Y. (2014). Cancer cell membrane-coated nanoparticles for anticancer vaccination and drug delivery. Nano Lett. 14 (4), 2181–2188. 10.1021/nl500618u 24673373 PMC3985711

[B59] Fang J. Islam W. Maeda H. (2020). Exploiting the dynamics of the EPR effect and strategies to improve the therapeutic effects of nanomedicines by using EPR effect enhancers. Adv. Drug Deliv. Rev. 157, 142–160. 10.1016/j.addr.2020.06.005 32553783

[B60] Fromen C. A. Fish M. B. Zimmerman A. Adili R. Holinstat M. Eniola-Adefeso O. (2016). Evaluation of receptor-ligand mechanisms of dual-targeted particles to an inflamed endothelium. Bioeng. Transl. Med. 1 (1), 103–115. 10.1002/btm2.10008 28066821 PMC5217161

[B61] Fu J. Li T. Yang Y. Jiang L. Wang W. Fu L. (2021). Activatable nanomedicine for overcoming hypoxia-induced resistance to chemotherapy and inhibiting tumor growth by inducing collaborative apoptosis and ferroptosis in solid tumors. Biomaterials 268, 120537. 10.1016/j.biomaterials.2020.120537 33260096

[B62] Gabizon A. Catane R. Uziely B. Kaufman B. Safra T. Cohen R. (1994). Prolonged circulation time and enhanced accumulation in malignant exudates of doxorubicin encapsulated in polyethylene-glycol coated liposomes. Cancer Res. 54 (4), 987–992. 8313389

[B63] Galstyan A. Markman J. L. Shatalova E. S. Chiechi A. Korman A. J. Patil R. (2019). Blood-brain barrier permeable nano immunoconjugates induce local immune responses for glioma therapy. Nat. Commun. 10 (1), 3850. 10.1038/s41467-019-11719-3 31462642 PMC6713723

[B64] Galvani S. Sanson M. Blaho V. A. Swendeman S. L. Obinata H. Conger H. (2015). HDL-bound sphingosine 1-phosphate acts as a biased agonist for the endothelial cell receptor S1P1 to limit vascular inflammation. Sci. Signal 8 (389), ra79. 10.1126/scisignal.aaa2581 26268607 PMC4768813

[B65] Gao J. Chu D. Wang Z. (2016). Cell membrane-formed nanovesicles for disease-targeted delivery. J. Control Release 224, 208–216. 10.1016/j.jconrel.2016.01.024 26778696 PMC4747686

[B66] Gao W. Li S. Liu Z. Sun Y. Cao W. Tong L. (2017). Targeting and destroying tumor vasculature with a near-infrared laser-activated "nanobomb" for efficient tumor ablation. Biomaterials 139, 1–11. 10.1016/j.biomaterials.2017.05.037 28578297

[B67] Gao X. Zhang J. Huang Z. Zuo T. Lu Q. Wu G. (2017). Reducing interstitial fluid pressure and inhibiting pulmonary metastasis of breast cancer by gelatin modified cationic lipid nanoparticles. ACS Appl. Mater Interfaces 9 (35), 29457–29468. 10.1021/acsami.7b05119 28799743

[B68] Gao W. Li X. Liu Z. Fu W. Sun Y. Cao W. (2019). A redox-responsive self-assembled nanoprobe for photoacoustic inflammation imaging to assess atherosclerotic plaque vulnerability. Anal. Chem. 91 (1), 1150–1156. 10.1021/acs.analchem.8b04912 30497260

[B69] Gao C. Huang Q. Liu C. Kwong C. H. T. Yue L. Wan J. B. (2020). Treatment of atherosclerosis by macrophage-biomimetic nanoparticles via targeted pharmacotherapy and sequestration of proinflammatory cytokines. Nat. Commun. 11 (1), 2622. 10.1038/s41467-020-16439-7 32457361 PMC7251120

[B70] Gao B. Xu J. Zhou J. Zhang H. Yang R. Wang H. (2021). Multifunctional pathology-mapping theranostic nanoplatforms for US/MR imaging and ultrasound therapy of atherosclerosis. Nanoscale 13 (18), 8623–8638. 10.1039/d1nr01096d 33929480

[B71] Ge C. Yang J. Duan S. Liu Y. Meng F. Yin L. (2020). Fluorinated α-helical polypeptides synchronize mucus permeation and cell penetration toward highly efficient pulmonary siRNA delivery against acute lung injury. Nano Lett. 20 (3), 1738–1746. 10.1021/acs.nanolett.9b04957 32039603

[B72] Gifani M. Eddins D. J. Kosuge H. Zhang Y. Paluri S. L. A. Larson T. (2021). Ultra-selective carbon nanotubes for photoacoustic imaging of inflamed atherosclerotic plaques. Adv. Funct. Mater 31 (37), 2101005. 10.1002/adfm.202101005 34733130 PMC8559995

[B73] Gimbrone M. A. Jr. García-Cardeña G. (2016). Endothelial cell dysfunction and the pathobiology of atherosclerosis. Circ. Res. 118 (4), 620–636. 10.1161/circresaha.115.306301 26892962 PMC4762052

[B74] Glicksman R. Chaudary N. Pintilie M. Leung E. Clarke B. Sy K. (2017). The predictive value of nadir neutrophil count during treatment of cervical cancer: Interactions with tumor hypoxia and interstitial fluid pressure (IFP). Clin. Transl. Radiat. Oncol. 6, 15–20. 10.1016/j.ctro.2017.08.002 29594218 PMC5862663

[B75] Golombek S. K. May J. N. Theek B. Appold L. Drude N. Kiessling F. (2018). Tumor targeting via EPR: Strategies to enhance patient responses. Adv. Drug Deliv. Rev. 130, 17–38. 10.1016/j.addr.2018.07.007 30009886 PMC6130746

[B76] Griesser J. Hetényi G. Federer C. Steinbring C. Ellemunter H. Niedermayr K. (2019). Highly mucus permeating and zeta potential changing self-emulsifying drug delivery systems: A potent gene delivery model for causal treatment of cystic fibrosis. Int. J. Pharm. 557, 124–134. 10.1016/j.ijpharm.2018.12.048 30594687

[B77] Gros A. Syvannarath V. Lamrani L. Ollivier V. Loyau S. Goerge T. (2015). Single platelets seal neutrophil-induced vascular breaches via GPVI during immune-complex-mediated inflammation in mice. Blood 126 (8), 1017–1026. 10.1182/blood-2014-12-617159 26036804

[B78] Guo H. Wang R. Wang D. Wang S. Zhou J. Chai Z. (2020). Deliver anti-PD-L1 into brain by p-hydroxybenzoic acid to enhance immunotherapeutic effect for glioblastoma. J. Control Release 320, 63–72. 10.1016/j.jconrel.2020.01.005 31917294

[B79] Guo Z. Hu Y. Zhao M. Hao K. He P. Tian H. (2021). Prodrug-based versatile nanomedicine with simultaneous physical and physiological tumor penetration for enhanced cancer chemo-immunotherapy. Nano Lett. 21 (9), 3721–3730. 10.1021/acs.nanolett.0c04772 33891423

[B80] Gupta S. Kesarla R. Chotai N. Misra A. Omri A. (2017). Systematic approach for the formulation and optimization of solid lipid nanoparticles of efavirenz by high pressure homogenization using design of experiments for brain targeting and enhanced bioavailability. Biomed. Res. Int. 2017, 1–18. 10.1155/2017/5984014 PMC5294220 28243600

[B81] Haber T. Cornejo Y. R. Aramburo S. Flores L. Cao P. Liu A. (2020). Specific targeting of ovarian tumor-associated macrophages by large, anionic nanoparticles. Proc. Natl. Acad. Sci. U. S. A. 117 (33), 19737–19745. 10.1073/pnas.1917424117 32732430 PMC7443897

[B82] Hansson G. K. (2005). Inflammation, atherosclerosis, and coronary artery disease. N. Engl. J. Med. 352 (16), 1685–1695. 10.1056/NEJMra043430 15843671

[B83] Hare J. I. Lammers T. Ashford M. B. Puri S. Storm G. Barry S. T. (2017). Challenges and strategies in anti-cancer nanomedicine development: An industry perspective. Adv. Drug Deliv. Rev. 108, 25–38. 10.1016/j.addr.2016.04.025 27137110

[B84] He Y. Wang M. Li X. Yu T. Gao X. (2020). Targeted MIP-3β plasmid nanoparticles induce dendritic cell maturation and inhibit M2 macrophage polarisation to suppress cancer growth. Biomaterials 249, 120046. 10.1016/j.biomaterials.2020.120046 32325346

[B85] He M. Qin Z. Liang X. He X. Zhu B. Lu Z. (2021). A pH-responsive mesoporous silica nanoparticles-based drug delivery system with controlled release of andrographolide for OA treatment. Regen. Biomater. 8 (4), rbab020. 10.1093/rb/rbab020 34221446 PMC8242227

[B86] Higashi T. Kogo T. Sato N. Hirotsu T. Misumi S. Nakamura H. (2020). Efficient anticancer drug delivery for pancreatic cancer treatment utilizing supramolecular polyethylene-glycosylated bromelain. ACS Appl. Bio Mater 3 (5), 3005–3014. 10.1021/acsabm.0c00070 35025347

[B87] Hillgruber C. Pöppelmann B. Weishaupt C. Steingräber A. K. Wessel F. Berdel W. E. (2015). Blocking neutrophil diapedesis prevents hemorrhage during thrombocytopenia. J. Exp. Med. 212 (8), 1255–1266. 10.1084/jem.20142076 26169941 PMC4516803

[B88] Hong S. Zheng D. W. Zhang C. Huang Q. X. Cheng S. X. Zhang X. Z. (2020). Vascular disrupting agent induced aggregation of gold nanoparticles for photothermally enhanced tumor vascular disruption. Sci. Adv. 6 (23), eabb0020. 10.1126/sciadv.abb0020 32548273 PMC7274768

[B89] Hori K. Saito S. Takahashi H. Sato H. Maeda H. Sato Y. (2000). Tumor-selective blood flow decrease induced by an angiotensin converting enzyme inhibitor, temocapril hydrochloride. Jpn. J. Cancer Res. 91 (2), 261–269. 10.1111/j.1349-7006.2000.tb00940.x 10761715 PMC5926331

[B90] Hoshyar N. Gray S. Han H. Bao G. (2016). The effect of nanoparticle size on *in vivo* pharmacokinetics and cellular interaction. Nanomedicine (Lond) 11 (6), 673–692. 10.2217/nnm.16.5 27003448 PMC5561790

[B91] Hou J. Zhou J. Chang M. Bao G. Xu J. Ye M. (2022). LIFU-responsive nanomedicine enables acoustic droplet vaporization-induced apoptosis of macrophages for stabilizing vulnerable atherosclerotic plaques. Bioact. Mater 16, 120–133. 10.1016/j.bioactmat.2022.02.022 35386311 PMC8958425

[B92] Hu C. M. Zhang L. Aryal S. Cheung C. Fang R. H. Zhang L. (2011). Erythrocyte membrane-camouflaged polymeric nanoparticles as a biomimetic delivery platform. Proc. Natl. Acad. Sci. U. S. A. 108 (27), 10980–10985. 10.1073/pnas.1106634108 21690347 PMC3131364

[B93] Hu C. M. Fang R. H. Wang K. C. Luk B. T. Thamphiwatana S. Dehaini D. (2015). Nanoparticle biointerfacing by platelet membrane cloaking. Nature 526 (7571), 118–121. 10.1038/nature15373 26374997 PMC4871317

[B94] Hu Q. Sun W. Qian C. Wang C. Bomba H. N. Gu Z. (2015). Anticancer platelet-mimicking nanovehicles. Adv. Mater 27 (44), 7043–7050. 10.1002/adma.201503323 26416431 PMC4998740

[B95] Hu Z. Ma J. Fu F. Cui C. Li X. Wang X. (2017). An intelligent re-shieldable targeting system for enhanced tumor accumulation. J. Control Release 268, 1–9. 10.1016/j.jconrel.2017.10.009 29030225

[B96] Hu J. K. Suh H. W. Qureshi M. Lewis J. M. Yaqoob S. Moscato Z. M. (2021). Nonsurgical treatment of skin cancer with local delivery of bioadhesive nanoparticles. Proc. Natl. Acad. Sci. U. S. A. 118 (7), e2020575118. 10.1073/pnas.2020575118 33526595 PMC7896333

[B97] Huang T. Zhao M. Yu Q. Feng Z. Xie M. Liu S. (2019). De novo design of polymeric carrier to photothermally release singlet oxygen for hypoxic tumor treatment. Res. (Wash D C) 2019, 9269081. 10.34133/2019/9269081 PMC6750110 31549095

[B98] Huckaby J. T. Lai S. K. (2018). PEGylation for enhancing nanoparticle diffusion in mucus. Adv. Drug Deliv. Rev. 124, 125–139. 10.1016/j.addr.2017.08.010 28882703

[B99] Infante J. R. Korn R. L. Rosen L. S. LoRusso P. Dychter S. S. Zhu J. (2018). Phase 1 trials of PEGylated recombinant human hyaluronidase PH20 in patients with advanced solid tumours. Br. J. Cancer 118 (2), 153–161. 10.1038/bjc.2017.327 28949957 PMC5785735

[B100] Jaaks P. Bernasconi M. (2017). The proprotein convertase furin in tumour progression. Int. J. Cancer 141 (4), 654–663. 10.1002/ijc.30714 28369813

[B101] Jahan S. T. Sadat S. M. A. Walliser M. Haddadi A. (2017). Targeted therapeutic nanoparticles: An immense promise to fight against cancer. J. Drug Deliv. 2017, 1–24. 10.1155/2017/9090325 PMC5804325 29464123

[B102] Jahanban-Esfahlan R. Seidi K. Zarghami N. (2017). Tumor vascular infarction: Prospects and challenges. Int. J. Hematol. 105 (3), 244–256. 10.1007/s12185-016-2171-3 28044258

[B103] Jain R. K. Stylianopoulos T. (2010). Delivering nanomedicine to solid tumors. Nat. Rev. Clin. Oncol. 7 (11), 653–664. 10.1038/nrclinonc.2010.139 20838415 PMC3065247

[B104] Jiang H. Hegde S. Knolhoff B. L. Zhu Y. Herndon J. M. Meyer M. A. (2016). Targeting focal adhesion kinase renders pancreatic cancers responsive to checkpoint immunotherapy. Nat. Med. 22 (8), 851–860. 10.1038/nm.4123 27376576 PMC4935930

[B105] Jiang N. Hu B. Cao S. Gao S. Cao Q. Chen J. (2020). Stable low-dose oxygen release using H(2)O(2)/perfluoropentane phase-change nanoparticles with low-intensity focused ultrasound for coronary thrombolysis. Ultrasound Med. Biol. 46 (10), 2765–2774. 10.1016/j.ultrasmedbio.2020.06.004 32646686

[B106] Jiang B. Jia X. Ji T. Zhou M. He J. Wang K. (2022). Ferritin nanocages for early theranostics of tumors via inflammation-enhanced active targeting. Sci. China Life Sci. 65 (2), 328–340. 10.1007/s11427-021-1976-0 34482518

[B107] Jin K. Luo Z. Zhang B. Pang Z. (2018). Biomimetic nanoparticles for inflammation targeting. Acta Pharm. Sin. B 8 (1), 23–33. 10.1016/j.apsb.2017.12.002 29872620 PMC5985691

[B108] Johnsen K. B. Burkhart A. Melander F. Kempen P. J. Vejlebo J. B. Siupka P. (2017). Targeting transferrin receptors at the blood-brain barrier improves the uptake of immunoliposomes and subsequent cargo transport into the brain parenchyma. Sci. Rep. 7 (1), 10396. 10.1038/s41598-017-11220-1 28871203 PMC5583399

[B109] Johnsen K. B. Bak M. Kempen P. J. Melander F. Burkhart A. Thomsen M. S. (2018). Antibody affinity and valency impact brain uptake of transferrin receptor-targeted gold nanoparticles. Theranostics 8 (12), 3416–3436. 10.7150/thno.25228 29930740 PMC6010987

[B110] Johnsen K. B. Bak M. Melander F. Thomsen M. S. Burkhart A. Kempen P. J. (2019). Modulating the antibody density changes the uptake and transport at the blood-brain barrier of both transferrin receptor-targeted gold nanoparticles and liposomal cargo. J. Control Release 295, 237–249. 10.1016/j.jconrel.2019.01.005 30633947

[B111] Kang L. J. Yoon J. Rho J. G. Han H. S. Lee S. Oh Y. S. (2021). Self-assembled hyaluronic acid nanoparticles for osteoarthritis treatment. Biomaterials 275, 120967. 10.1016/j.biomaterials.2021.120967 34153786

[B112] Kelley W. J. Safari H. Lopez-Cazares G. Eniola-Adefeso O. (2016). Vascular-targeted nanocarriers: Design considerations and strategies for successful treatment of atherosclerosis and other vascular diseases. Wiley Interdiscip. Rev. Nanomed Nanobiotechnol. 8 (6), 909–926. 10.1002/wnan.1414 27194461 PMC5065366

[B113] Kim K. S. Youn Y. S. Bae Y. H. (2019). Immune-triggered cancer treatment by intestinal lymphatic delivery of docetaxel-loaded nanoparticle. J. Control Release 311-312, 85–95. 10.1016/j.jconrel.2019.08.027 31461664

[B114] Kim Y. Uthaman S. Pillarisetti S. Noh K. Huh K. M. Park I. K. (2020). Bioactivatable reactive oxygen species-sensitive nanoparticulate system for chemo-photodynamic therapy. Acta Biomater. 108, 273–284. 10.1016/j.actbio.2020.03.027 32205212

[B115] Kinnear C. Moore T. L. Rodriguez-Lorenzo L. Rothen-Rutishauser B. Petri-Fink A. (2017). Form follows function: Nanoparticle shape and its implications for nanomedicine. Chem. Rev. 117 (17), 11476–11521. 10.1021/acs.chemrev.7b00194 28862437

[B116] Knudson W. Ishizuka S. Terabe K. Askew E. B. Knudson C. B. (2019). The pericellular hyaluronan of articular chondrocytes. Matrix Biol. 78-79, 32–46. 10.1016/j.matbio.2018.02.005 29425696 PMC6078830

[B117] Koczera P. Appold L. Shi Y. Liu M. Dasgupta A. Pathak V. (2017). PBCA-based polymeric microbubbles for molecular imaging and drug delivery. J. Control Release 259, 128–135. 10.1016/j.jconrel.2017.03.006 28279799 PMC5528138

[B118] Kodama H. Shamay Y. Kimura Y. Shah J. Solomon S. B. Heller D. (2019). Electroporation-induced changes in tumor vasculature and microenvironment can promote the delivery and increase the efficacy of sorafenib nanoparticles. Bioelectrochemistry 130, 107328. 10.1016/j.bioelechem.2019.107328 31306879 PMC6859646

[B119] Kolaczkowska E. Kubes P. (2013). Neutrophil recruitment and function in health and inflammation. Nat. Rev. Immunol. 13 (3), 159–175. 10.1038/nri3399 23435331

[B120] Kou L. Bhutia Y. D. Yao Q. He Z. Sun J. Ganapathy V. (2018). Transporter-Guided delivery of nanoparticles to improve drug permeation across cellular barriers and drug exposure to selective cell types. Front. Pharmacol. 9, 27. 10.3389/fphar.2018.00027 29434548 PMC5791163

[B121] Kunde S. S. Wairkar S. (2021). Platelet membrane camouflaged nanoparticles: Biomimetic architecture for targeted therapy. Int. J. Pharm. 598, 120395. 10.1016/j.ijpharm.2021.120395 33639226

[B122] Kuzmov A. Minko T. (2015). Nanotechnology approaches for inhalation treatment of lung diseases. J. Control Release 219, 500–518. 10.1016/j.jconrel.2015.07.024 26297206

[B123] Lamson N. G. Berger A. Fein K. C. Whitehead K. A. (2020). Anionic nanoparticles enable the oral delivery of proteins by enhancing intestinal permeability. Nat. Biomed. Eng. 4 (1), 84–96. 10.1038/s41551-019-0465-5 31686002 PMC7461704

[B124] Lapin N. A. Gill K. Shah B. R. Chopra R. (2020). Consistent opening of the blood brain barrier using focused ultrasound with constant intravenous infusion of microbubble agent. Sci. Rep. 10 (1), 16546. 10.1038/s41598-020-73312-9 33024157 PMC7538995

[B125] Lapteva M. Mondon K. Möller M. Gurny R. Kalia Y. N. (2014). Polymeric micelle nanocarriers for the cutaneous delivery of tacrolimus: A targeted approach for the treatment of psoriasis. Mol. Pharm. 11 (9), 2989–3001. 10.1021/mp400639e 25057896

[B126] Lee W. L. Slutsky A. S. (2010). Sepsis and endothelial permeability. N. Engl. J. Med. 363 (7), 689–691. 10.1056/NEJMcibr1007320 20818861

[B127] Lee S. Han H. Koo H. Na J. H. Yoon H. Y. Lee K. E. (2017). Extracellular matrix remodeling *in vivo* for enhancing tumor-targeting efficiency of nanoparticle drug carriers using the pulsed high intensity focused ultrasound. J. Control Release 263, 68–78. 10.1016/j.jconrel.2017.02.035 28257990

[B128] Li S. Zhang Y. Wang J. Zhao Y. Ji T. Zhao X. (2017). Nanoparticle-mediated local depletion of tumour-associated platelets disrupts vascular barriers and augments drug accumulation in tumours. Nat. Biomed. Eng. 1 (8), 667–679. 10.1038/s41551-017-0115-8 31015598

[B129] Li Z. Zhang Y. Zhu D. Li S. Yu X. Zhao Y. (2017). Transporting carriers for intracellular targeting delivery via non-endocytic uptake pathways. Drug Deliv. 24, 45–55. 10.1080/10717544.2017.1391889 29069996 PMC8812582

[B130] Li X. Cao C. Wei P. Xu M. Liu Z. Liu L. (2019). Self-assembly of amphiphilic peptides for recognizing high furin-expressing cancer cells. ACS Appl. Mater Interfaces 11 (13), 12327–12334. 10.1021/acsami.9b01281 30864434

[B131] Li G. Wang S. Deng D. Xiao Z. Dong Z. Wang Z. (2020). Fluorinated chitosan to enhance transmucosal delivery of sonosensitizer-conjugated catalase for sonodynamic bladder cancer treatment post-intravesical instillation. ACS Nano 14 (2), 1586–1599. 10.1021/acsnano.9b06689 32011860

[B132] Li S. Zhang Y. Ho S. H. Li B. Wang M. Deng X. (2020b). Combination of tumour-infarction therapy and chemotherapy via the co-delivery of doxorubicin and thrombin encapsulated in tumour-targeted nanoparticles. Nat. Biomed. Eng. 4 (7), 732–742. 10.1038/s41551-020-0573-2 32572197

[B133] Li H. Shi S. Wu M. Shen W. Ren J. Mei Z. (2021a). iRGD peptide-mediated liposomal nanoparticles with photoacoustic/ultrasound dual-modality imaging for precision theranostics against hepatocellular carcinoma. Int. J. Nanomed. 16, 6455–6475. 10.2147/ijn.S325891 PMC8464346 34584411

[B134] Li J. Zheng M. Shimoni O. Banks W. A. Bush A. I. Gamble J. R. (2021b). Development of novel therapeutics targeting the blood-brain barrier: From barrier to carrier. Adv. Sci. (Weinh) 8 (16), e2101090. 10.1002/advs.202101090 34085418 PMC8373165

[B135] Li X. Vemireddy V. Cai Q. Xiong H. Kang P. Li X. (2021c). Reversibly modulating the blood-brain barrier by laser stimulation of molecular-targeted nanoparticles. Nano Lett. 21 (22), 9805–9815. 10.1021/acs.nanolett.1c02996 34516144 PMC8616836

[B136] Li D. F. Yang M. F. Xu H. M. Zhu M. Z. Zhang Y. Tian C. M. (2022). Nanoparticles for oral delivery: Targeted therapy for inflammatory bowel disease. J. Mater Chem. B 10 (31), 5853–5872. 10.1039/d2tb01190e 35876136

[B137] Liang X. Li H. Zhang A. Tian X. Guo H. Zhang H. (2022). Red blood cell biomimetic nanoparticle with anti-inflammatory, anti-oxidative and hypolipidemia effect ameliorated atherosclerosis therapy. Nanomedicine 41, 102519. 10.1016/j.nano.2022.102519 35038590

[B138] Libby P. Ridker P. M. Maseri A. (2002). Inflammation and atherosclerosis. Circulation 105 (9), 1135–1143. 10.1161/hc0902.104353 11877368

[B139] Lin C. Tong F. Liu R. Xie R. Lei T. Chen Y. (2020). GSH-responsive SN38 dimer-loaded shape-transformable nanoparticles with iRGD for enhancing chemo-photodynamic therapy. Acta Pharm. Sin. B 10 (12), 2348–2361. 10.1016/j.apsb.2020.10.009 33354506 PMC7745177

[B140] Liu X. Lin P. Perrett I. Lin J. Liao Y. P. Chang C. H. (2017). Tumor-penetrating peptide enhances transcytosis of silicasome-based chemotherapy for pancreatic cancer. J. Clin. Invest. 127 (5), 2007–2018. 10.1172/jci92284 28414297 PMC5409788

[B141] Liu R. Xiao W. Hu C. Xie R. Gao H. (2018). Theranostic size-reducible and no donor conjugated gold nanocluster fabricated hyaluronic acid nanoparticle with optimal size for combinational treatment of breast cancer and lung metastasis. J. Control Release 278, 127–139. 10.1016/j.jconrel.2018.04.005 29630985

[B142] Liu H. Jiang W. Wang Q. Xia J. Yu W. Wang Y. (2020). Microenvironment-activated nanoparticles for oxygen self-supplemented photodynamic cancer therapy. Biomater. Sci. 8 (1), 370–378. 10.1039/c9bm01537j 31728482

[B143] Liu B. Yan W. Luo L. Wu S. Wang Y. Zhong Y. (2021). Macrophage membrane camouflaged reactive oxygen species responsive nanomedicine for efficiently inhibiting the vascular intimal hyperplasia. J. Nanobiotechnol. 19 (1), 374. 10.1186/s12951-021-01119-5 PMC8600790 34789284

[B144] Liu Y. Xu L. Zhang Q. Kang Y. Liu L. Liu Z. (2022). Localized myocardial anti-inflammatory effects of temperature-sensitive budesonide nanoparticles during radiofrequency catheter ablation. Res. (Wash D C) 2022, 9816234. 10.34133/2022/9816234 PMC9178488 35707046

[B145] Lockhart J. H. VanWye J. Banerjee R. Wickline S. A. Pan H. Totary-Jain H. (2021). Self-assembled miRNA-switch nanoparticles target denuded regions and prevent restenosis. Mol. Ther. 29 (5), 1744–1757. 10.1016/j.ymthe.2021.01.032 33545360 PMC8116603

[B146] Lou H. Ji A. Qu C. Liu H. Jiang L. Chen H. (2022). A small-molecule based organic nanoparticle for photothermal therapy and near-infrared-IIb imaging. ACS Appl. Mater Interfaces 14 (31), 35454–35465. 10.1021/acsami.2c11706 35900924

[B147] Luk B. T. Hu C. M. Fang R. H. Dehaini D. Carpenter C. Gao W. (2014). Interfacial interactions between natural RBC membranes and synthetic polymeric nanoparticles. Nanoscale 6 (5), 2730–2737. 10.1039/c3nr06371b 24463706 PMC3954976

[B148] Lundy D. J. Chen K. H. Toh E. K. Hsieh P. C. (2016). Distribution of systemically administered nanoparticles reveals a size-dependent effect immediately following cardiac ischaemia-reperfusion injury. Sci. Rep. 6, 25613. 10.1038/srep25613 27161857 PMC4861966

[B149] Ma L. Wang Y. Zhang S. Qian X. Xue N. Jiang Z. (2020). Deep penetration of targeted nanobubbles enhanced cavitation effect on thrombolytic capacity. Bioconjug Chem. 31 (2), 369–374. 10.1021/acs.bioconjchem.9b00653 31765569

[B150] Maeda H. Nakamura H. Fang J. (2013). The EPR effect for macromolecular drug delivery to solid tumors: Improvement of tumor uptake, lowering of systemic toxicity, and distinct tumor imaging *in vivo* . Adv. Drug Deliv. Rev. 65 (1), 71–79. 10.1016/j.addr.2012.10.002 23088862

[B151] Mai Y. Ouyang Y. Yu M. Qin Y. Girardi M. Saltzman W. M. (2022). Topical formulation based on disease-specific nanoparticles for single-dose cure of psoriasis. J. Control Release 349, 354–366. 10.1016/j.jconrel.2022.07.006 35817278

[B152] Mangal S. Gao W. Li T. Zhou Q. T. (2017). Pulmonary delivery of nanoparticle chemotherapy for the treatment of lung cancers: Challenges and opportunities. Acta Pharmacol. Sin. 38 (6), 782–797. 10.1038/aps.2017.34 28504252 PMC5520191

[B153] Mantovani A. Marchesi F. Malesci A. Laghi L. Allavena P. (2017). Tumour-associated macrophages as treatment targets in oncology. Nat. Rev. Clin. Oncol. 14 (7), 399–416. 10.1038/nrclinonc.2016.217 28117416 PMC5480600

[B154] Mao L. Wu W. Wang M. Guo J. Li H. Zhang S. (2021). Targeted treatment for osteoarthritis: Drugs and delivery system. Drug Deliv. 28 (1), 1861–1876. 10.1080/10717544.2021.1971798 34515606 PMC8439249

[B155] Martinez J. O. Molinaro R. Hartman K. A. Boada C. Sukhovershin R. De Rosa E. (2018). Biomimetic nanoparticles with enhanced affinity towards activated endothelium as versatile tools for theranostic drug delivery. Theranostics 8 (4), 1131–1145. 10.7150/thno.22078 29464004 PMC5817115

[B156] Martínez-Jothar L. Barendrecht A. D. de Graaff A. M. Oliveira S. van Nostrum C. F. Schiffelers R. M. (2020). Endothelial cell targeting by cRGD-functionalized polymeric nanoparticles under static and flow conditions. Nanomater. (Basel) 10 (7), 1353. 10.3390/nano10071353 PMC7407316 32664364

[B157] Matsumura Y. Maeda H. (1986). A new concept for macromolecular therapeutics in cancer chemotherapy: Mechanism of tumoritropic accumulation of proteins and the antitumor agent smancs. Cancer Res. 46, 6387–6392. 2946403

[B158] Matthay M. A. Ware L. B. Zimmerman G. A. (2012). The acute respiratory distress syndrome. J. Clin. Invest. 122 (8), 2731–2740. 10.1172/jci60331 22850883 PMC3408735

[B159] Miguel R. D. A. Hirata A. S. Jimenez P. C. Lopes L. B. Costa-Lotufo L. V. (2022). Beyond formulation: Contributions of nanotechnology for translation of anticancer natural products into new drugs. Pharmaceutics 14 (8), 1722. 10.3390/pharmaceutics14081722 36015347 PMC9415580

[B160] Min Y. Caster J. M. Eblan M. J. Wang A. Z. (2015). Clinical translation of nanomedicine. Chem. Rev. 115 (19), 11147–11190. 10.1021/acs.chemrev.5b00116 26088284 PMC4607605

[B161] Miyazawa B. Trivedi A. Togarrati P. P. Potter D. Baimukanova G. Vivona L. (2019). Regulation of endothelial cell permeability by platelet-derived extracellular vesicles. J. Trauma Acute Care Surg. 86 (6), 931–942. 10.1097/ta.0000000000002230 31124890 PMC7381393

[B162] Mo J. Xie Q. Wei W. Zhao J. (2018). Revealing the immune perturbation of black phosphorus nanomaterials to macrophages by understanding the protein corona. Nat. Commun. 9 (1), 2480. 10.1038/s41467-018-04873-7 29946125 PMC6018659

[B163] Mohideen M. Quijano E. Song E. Deng Y. Panse G. Zhang W. (2017). Degradable bioadhesive nanoparticles for prolonged intravaginal delivery and retention of elvitegravir. Biomaterials 144, 144–154. 10.1016/j.biomaterials.2017.08.029 28829952 PMC5581224

[B164] Molinaro R. Evangelopoulos M. Hoffman J. R. Corbo C. Taraballi F. Martinez J. O. (2018). Design and development of biomimetic nanovesicles using a microfluidic approach. Adv. Mater 30 (15), e1702749. 10.1002/adma.201702749 29512198

[B165] Molloy C. P. Yao Y. Kammoun H. Bonnard T. Hoefer T. Alt K. (2017). Shear-sensitive nanocapsule drug release for site-specific inhibition of occlusive thrombus formation. J. Thromb. Haemost. 15 (5), 972–982. 10.1111/jth.13666 28267256

[B166] Moyano D. F. Liu Y. Peer D. Rotello V. M. (2016). Modulation of immune response using engineered nanoparticle surfaces. Small 12 (1), 76–82. 10.1002/smll.201502273 26618755 PMC4749139

[B167] Nag O. K. Delehanty J. B. (2019). Active cellular and subcellular targeting of nanoparticles for drug delivery. Pharmaceutics 11 (10), 543. 10.3390/pharmaceutics11100543 31635367 PMC6836276

[B168] Nakamura Y. Mochida A. Choyke P. L. Kobayashi H. (2016). Nanodrug delivery: Is the enhanced permeability and retention effect sufficient for curing cancer? Bioconjug Chem. 27 (10), 2225–2238. 10.1021/acs.bioconjchem.6b00437 27547843 PMC7397928

[B169] Nakashima Y. Raines E. W. Plump A. S. Breslow J. L. Ross R. (1998). Upregulation of VCAM-1 and ICAM-1 at atherosclerosis-prone sites on the endothelium in the ApoE-deficient mouse. Arterioscler. Thromb. Vasc. Biol. 18 (5), 842–851. 10.1161/01.atv.18.5.842 9598845

[B170] Narain A. Asawa S. Chhabria V. Patil-Sen Y. (2017). Cell membrane coated nanoparticles: Next-generation therapeutics. Nanomedicine (Lond) 12 (21), 2677–2692. 10.2217/nnm-2017-0225 28965474

[B171] Ndayishimiye J. Cao Y. Kumeria T. Blaskovich M. A. T. Falconer J. R. Popat A. (2021). Engineering mesoporous silica nanoparticles towards oral delivery of vancomycin. J. Mater Chem. B 9 (35), 7145–7166. 10.1039/d1tb01430g 34525166

[B172] Nel A. Xia T. Mädler L. Li N. (2006). Toxic potential of materials at the nanolevel. Science 311 (5761), 622–627. 10.1126/science.1114397 16456071

[B173] Nissen N. I. Karsdal M. Willumsen N. (2019). Collagens and Cancer associated fibroblasts in the reactive stroma and its relation to Cancer biology. J. Exp. Clin. Cancer Res. 38 (1), 115. 10.1186/s13046-019-1110-6 30841909 PMC6404286

[B174] Niu W. Xiao Q. Wang X. Zhu J. Li J. Liang X. (2021). A biomimetic drug delivery system by integrating grapefruit extracellular vesicles and doxorubicin-loaded heparin-based nanoparticles for glioma therapy. Nano Lett. 21 (3), 1484–1492. 10.1021/acs.nanolett.0c04753 33475372

[B175] Ojha T. Pathak V. Shi Y. Hennink W. E. Moonen C. T. W. Storm G. (2017). Pharmacological and physical vessel modulation strategies to improve EPR-mediated drug targeting to tumors. Adv. Drug Deliv. Rev. 119, 44–60. 10.1016/j.addr.2017.07.007 28697952 PMC5919100

[B176] Olsman M. Sereti V. Andreassen K. Snipstad S. van Wamel A. Eliasen R. (2020). Ultrasound-mediated delivery enhances therapeutic efficacy of MMP sensitive liposomes. J. Control Release 325, 121–134. 10.1016/j.jconrel.2020.06.024 32621827

[B177] Olsman M. Sereti V. Mühlenpfordt M. Johnsen K. B. Andresen T. L. Urquhart A. J. (2021). Focused ultrasound and microbubble treatment increases delivery of transferrin receptor-targeting liposomes to the brain. Ultrasound Med. Biol. 47 (5), 1343–1355. 10.1016/j.ultrasmedbio.2021.01.014 33608142

[B178] Osman G. Rodriguez J. Chan S. Y. Chisholm J. Duncan G. Kim N. (2018). PEGylated enhanced cell penetrating peptide nanoparticles for lung gene therapy. J. Control Release 285, 35–45. 10.1016/j.jconrel.2018.07.001 30004000 PMC6573017

[B179] Osman N. M. Sexton D. W. Saleem I. Y. (2020). Toxicological assessment of nanoparticle interactions with the pulmonary system. Nanotoxicology 14 (1), 21–58. 10.1080/17435390.2019.1661043 31502904

[B180] Paiva I. Mattingly S. Wuest M. Leier S. Vakili M. R. Weinfeld M. (2020). Synthesis and analysis of (64)Cu-labeled GE11-modified polymeric micellar nanoparticles for EGFR-targeted molecular imaging in a colorectal cancer model. Mol. Pharm. 17 (5), 1470–1481. 10.1021/acs.molpharmaceut.9b01043 32233491

[B181] Pandit S. Dutta D. Nie S. (2020). Active transcytosis and new opportunities for cancer nanomedicine. Nat. Mater 19 (5), 478–480. 10.1038/s41563-020-0672-1 32332990

[B182] Paris J. L. Villaverde G. Gómez-Graña S. Vallet-Regí M. (2020). Nanoparticles for multimodal antivascular therapeutics: Dual drug release, photothermal and photodynamic therapy. Acta Biomater. 101, 459–468. 10.1016/j.actbio.2019.11.004 31706040 PMC7616912

[B183] Parvathaneni V. Shukla S. K. Kulkarni N. S. Gupta V. (2021). Development and characterization of inhalable transferrin functionalized amodiaquine nanoparticles - efficacy in Non-Small Cell Lung Cancer (NSCLC) treatment. Int. J. Pharm. 608, 121038. 10.1016/j.ijpharm.2021.121038 34438008

[B184] Patra J. K. Das G. Fraceto L. F. Campos E. V. R. Rodriguez-Torres M. D. P. Acosta-Torres L. S. (2018). Nano based drug delivery systems: Recent developments and future prospects. J. Nanobiotechnol. 16 (1), 71. 10.1186/s12951-018-0392-8 PMC6145203 30231877

[B185] Pinheiro R. G. R. Coutinho A. J. Pinheiro M. Neves A. R. (2021). Nanoparticles for targeted brain drug delivery: What do we know? Int. J. Mol. Sci. 22 (21), 11654. 10.3390/ijms222111654 34769082 PMC8584083

[B186] Pober J. S. Sessa W. C. (2007). Evolving functions of endothelial cells in inflammation. Nat. Rev. Immunol. 7 (10), 803–815. 10.1038/nri2171 17893694

[B187] Poltavets V. Kochetkova M. Pitson S. M. Samuel M. S. (2018). The role of the extracellular matrix and its molecular and cellular regulators in cancer cell plasticity. Front. Oncol. 8, 431. 10.3389/fonc.2018.00431 30356678 PMC6189298

[B188] Rafii S. Butler J. M. Ding B. S. (2016). Angiocrine functions of organ-specific endothelial cells. Nature 529 (7586), 316–325. 10.1038/nature17040 26791722 PMC4878406

[B189] Ren H. He Y. Liang J. Cheng Z. Zhang M. Zhu Y. (2019). Role of liposome size, surface charge, and PEGylation on rheumatoid arthritis targeting therapy. ACS Appl. Mater Interfaces 11 (22), 20304–20315. 10.1021/acsami.8b22693 31056910

[B190] Ridker P. M. Everett B. M. Thuren T. MacFadyen J. G. Chang W. H. Ballantyne C. (2017). Antiinflammatory therapy with canakinumab for atherosclerotic disease. N. Engl. J. Med. 377 (12), 1119–1131. 10.1056/NEJMoa1707914 28845751

[B191] Rosière R. Van Woensel M. Gelbcke M. Mathieu V. Hecq J. Mathivet T. (2018). New folate-grafted chitosan derivative to improve delivery of paclitaxel-loaded solid lipid nanoparticles for lung tumor therapy by inhalation. Mol. Pharm. 15 (3), 899–910. 10.1021/acs.molpharmaceut.7b00846 29341619

[B192] Ruan G. Ye L. Liu G. An J. Sehouli J. Sun P. (2018). The role of bevacizumab in targeted vascular endothelial growth factor therapy for epithelial ovarian cancer: An updated systematic review and meta-analysis. Onco Targets Ther. 11, 521–528. 10.2147/ott.S155581 29416352 PMC5788992

[B193] Sala M. Diab R. Elaissari A. Fessi H. (2018). Lipid nanocarriers as skin drug delivery systems: Properties, mechanisms of skin interactions and medical applications. Int. J. Pharm. 535 (1-2), 1–17. 10.1016/j.ijpharm.2017.10.046 29111097

[B194] Salatin S. Yari Khosroushahi A. (2017). Overviews on the cellular uptake mechanism of polysaccharide colloidal nanoparticles. J. Cell Mol. Med. 21 (9), 1668–1686. 10.1111/jcmm.13110 28244656 PMC5571529

[B195] Scaranti M. Cojocaru E. Banerjee S. Banerji U. (2020). Exploiting the folate receptor α in oncology. Nat. Rev. Clin. Oncol. 17 (6), 349–359. 10.1038/s41571-020-0339-5 32152484

[B196] Schneider C. S. Xu Q. Boylan N. J. Chisholm J. Tang B. C. Schuster B. S. (2017). Nanoparticles that do not adhere to mucus provide uniform and long-lasting drug delivery to airways following inhalation. Sci. Adv. 3 (4), e1601556. 10.1126/sciadv.1601556 28435870 PMC5381952

[B197] Seong J. Wang N. Wang Y. (2013). Mechanotransduction at focal adhesions: From physiology to cancer development. J. Cell Mol. Med. 17 (5), 597–604. 10.1111/jcmm.12045 23601032 PMC3665742

[B198] Serrels A. Lund T. Serrels B. Byron A. McPherson R. C. von Kriegsheim A. (2015). Nuclear FAK controls chemokine transcription, Tregs, and evasion of anti-tumor immunity. Cell 163 (1), 160–173. 10.1016/j.cell.2015.09.001 26406376 PMC4597190

[B199] Shah R. Patel T. Freedman J. E. (2018). Circulating extracellular vesicles in human disease. N. Engl. J. Med. 379 (10), 958–966. 10.1056/NEJMra1704286 30184457

[B200] Shen A. M. Minko T. (2020). Pharmacokinetics of inhaled nanotherapeutics for pulmonary delivery. J. Control Release 326, 222–244. 10.1016/j.jconrel.2020.07.011 32681948 PMC7501141

[B201] Shen M. Y. Liu T. I. Yu T. W. Kv R. Chiang W. H. Tsai Y. C. (2019). Hierarchically targetable polysaccharide-coated solid lipid nanoparticles as an oral chemo/thermotherapy delivery system for local treatment of colon cancer. Biomaterials 197, 86–100. 10.1016/j.biomaterials.2019.01.019 30641267

[B202] Shi Q. Zhang Y. Liu S. Liu G. Xu J. Zhao X. (2018). Specific tissue factor delivery using a tumor-homing peptide for inducing tumor infarction. Biochem. Pharmacol. 156, 501–510. 10.1016/j.bcp.2018.09.020 30222966

[B203] Shim M. K. Park J. Yoon H. Y. Lee S. Um W. Kim J. H. (2019). Carrier-free nanoparticles of cathepsin B-cleavable peptide-conjugated doxorubicin prodrug for cancer targeting therapy. J. Control Release 294, 376–389. 10.1016/j.jconrel.2018.11.032 30550940

[B204] Sigismund S. Avanzato D. Lanzetti L. (2018). Emerging functions of the EGFR in cancer. Mol. Oncol. 12 (1), 3–20. 10.1002/1878-0261.12155 29124875 PMC5748484

[B205] Silverman J. A. Deitcher S. R. (2013). Marqibo® (vincristine sulfate liposome injection) improves the pharmacokinetics and pharmacodynamics of vincristine. Cancer Chemother. Pharmacol. 71 (3), 555–564. 10.1007/s00280-012-2042-4 23212117 PMC3579462

[B206] Sindhwani S. Syed A. M. Ngai J. Kingston B. R. Maiorino L. Rothschild J. (2020). The entry of nanoparticles into solid tumours. Nat. Mater 19 (5), 566–575. 10.1038/s41563-019-0566-2 31932672

[B207] Soe Z. C. Kwon J. B. Thapa R. K. Ou W. Nguyen H. T. Gautam M. (2019). Transferrin-conjugated polymeric nanoparticle for receptor-mediated delivery of doxorubicin in doxorubicin-resistant breast cancer cells. Pharmaceutics 11 (2), 63. 10.3390/pharmaceutics11020063 30717256 PMC6410246

[B208] Song E. Gaudin A. King A. R. Seo Y. E. Suh H. W. Deng Y. (2017). Surface chemistry governs cellular tropism of nanoparticles in the brain. Nat. Commun. 8, 15322. 10.1038/ncomms15322 28524852 PMC5454541

[B209] Song Y. Huang Z. Liu X. Pang Z. Chen J. Yang H. (2019). Platelet membrane-coated nanoparticle-mediated targeting delivery of Rapamycin blocks atherosclerotic plaque development and stabilizes plaque in apolipoprotein E-deficient (ApoE(-/-)) mice. Nanomedicine 15 (1), 13–24. 10.1016/j.nano.2018.08.002 30171903

[B210] Song Y. Li D. Lu Y. Jiang K. Yang Y. Xu Y. (2020). Ferrimagnetic mPEG-b-PHEP copolymer micelles loaded with iron oxide nanocubes and emodin for enhanced magnetic hyperthermia-chemotherapy. Natl. Sci. Rev. 7 (4), 723–736. 10.1093/nsr/nwz201 34692091 PMC8289054

[B211] Sprague D. L. Sowa J. M. Elzey B. D. Ratliff T. L. (2007). The role of platelet CD154 in the modulation in adaptive immunity. Immunol. Res. 39 (1-3), 185–193. 10.1007/s12026-007-0074-3 17917065

[B212] Srimathveeravalli G. Abdel-Atti D. Pérez-Medina C. Takaki H. Solomon S. B. Mulder W. J. M. (2018). Reversible electroporation-mediated liposomal doxorubicin delivery to tumors can Be monitored with (89)Zr-labeled reporter nanoparticles. Mol. Imaging 17, 153601211774972. 10.1177/1536012117749726 PMC5833236 29480077

[B213] Sudhakar S. Chandran S. V. Selvamurugan N. Nazeer R. A. (2020). Biodistribution and pharmacokinetics of thiolated chitosan nanoparticles for oral delivery of insulin *in vivo* . Int. J. Biol. Macromol. 150, 281–288. 10.1016/j.ijbiomac.2020.02.079 32057846

[B214] Suk J. S. Xu Q. Kim N. Hanes J. Ensign L. M. (2016). PEGylation as a strategy for improving nanoparticle-based drug and gene delivery. Adv. Drug Deliv. Rev. 99, 28–51. 10.1016/j.addr.2015.09.012 26456916 PMC4798869

[B215] Sun T. Dasgupta A. Zhao Z. Nurunnabi M. Mitragotri S. (2020). Physical triggering strategies for drug delivery. Adv. Drug Deliv. Rev. 158, 36–62. 10.1016/j.addr.2020.06.010 32589905

[B216] Ta H. T. Truong N. P. Whittaker A. K. Davis T. P. Peter K. (2018). The effects of particle size, shape, density and flow characteristics on particle margination to vascular walls in cardiovascular diseases. Expert Opin. Drug Deliv. 15 (1), 33–45. 10.1080/17425247.2017.1316262 28388248

[B217] Tabatabaei S. N. Girouard H. Carret A. S. Martel S. (2015). Remote control of the permeability of the blood-brain barrier by magnetic heating of nanoparticles: A proof of concept for brain drug delivery. J. Control Release 206, 49–57. 10.1016/j.jconrel.2015.02.027 25724273

[B218] Tanaka N. Kanatani S. Tomer R. Sahlgren C. Kronqvist P. Kaczynska D. (2017). Whole-tissue biopsy phenotyping of three-dimensional tumours reveals patterns of cancer heterogeneity. Nat. Biomed. Eng. 1 (10), 796–806. 10.1038/s41551-017-0139-0 31015588

[B219] Terstappen G. C. Meyer A. H. Bell R. D. Zhang W. (2021). Strategies for delivering therapeutics across the blood-brain barrier. Nat. Rev. Drug Discov. 20 (5), 362–383. 10.1038/s41573-021-00139-y 33649582

[B220] Thavarajah R. Mudimbaimannar V. K. Elizabeth J. Rao U. K. Ranganathan K. (2012). Chemical and physical basics of routine formaldehyde fixation. J. Oral Maxillofac. Pathol. 16 (3), 400–405. 10.4103/0973-029x.102496 23248474 PMC3519217

[B221] Trac N. Chen L. Y. Zhang A. Liao C. P. Poon C. Wang J. (2021). CCR2-targeted micelles for anti-cancer peptide delivery and immune stimulation. J. Control Release 329, 614–623. 10.1016/j.jconrel.2020.09.054 33011241 PMC8491563

[B222] Tu Z. Zhong Y. Hu H. Shao D. Haag R. Schirner M. (2022). Design of therapeutic biomaterials to control inflammation. Nat. Rev. Mater 7 (7), 557–574. 10.1038/s41578-022-00426-z 35251702 PMC8884103

[B223] Uthaman S. Huh K. M. Park I. K. (2018). Tumor microenvironment-responsive nanoparticles for cancer theragnostic applications. Biomater. Res. 22, 22. 10.1186/s40824-018-0132-z 30155269 PMC6108142

[B224] Vemula S. Shi J. Hanneman P. Wei L. Kapur R. (2010). ROCK1 functions as a suppressor of inflammatory cell migration by regulating PTEN phosphorylation and stability. Blood 115 (9), 1785–1796. 10.1182/blood-2009-08-237222 20008297 PMC2832812

[B225] Villaseñor R. Lampe J. Schwaninger M. Collin L. (2019). Intracellular transport and regulation of transcytosis across the blood-brain barrier. Cell Mol. Life Sci. 76 (6), 1081–1092. 10.1007/s00018-018-2982-x 30523362 PMC6513804

[B226] Wagner S. C. Ichim T. E. Ma H. Szymanski J. Perez J. A. Lopez J. (2015). Cancer anti-angiogenesis vaccines: Is the tumor vasculature antigenically unique? J. Transl. Med. 13, 340. 10.1186/s12967-015-0688-5 26510973 PMC4625691

[B227] Wan F. Herzberg M. Huang Z. Hassenkam T. Nielsen H. M. (2020). A free-floating mucin layer to investigate the effect of the local microenvironment in lungs on mucin-nanoparticle interactions. Acta Biomater. 104, 115–123. 10.1016/j.actbio.2020.01.014 31945503

[B228] Wang S. Shin I. S. Hancock H. Jang B. S. Kim H. S. Lee S. M. (2012). Pulsed high intensity focused ultrasound increases penetration and therapeutic efficacy of monoclonal antibodies in murine xenograft tumors. J. Control Release 162 (1), 218–224. 10.1016/j.jconrel.2012.06.025 22732476 PMC4219504

[B229] Wang Q. Ren Y. Mu J. Egilmez N. K. Zhuang X. Deng Z. (2015). Grapefruit-derived nanovectors use an activated leukocyte trafficking pathway to deliver therapeutic agents to inflammatory tumor sites. Cancer Res. 75 (12), 2520–2529. 10.1158/0008-5472.Can-14-3095 25883092 PMC4470740

[B230] Wang Y. Zhang K. Qin X. Li T. Qiu J. Yin T. (2019). Biomimetic nanotherapies: Red blood cell based core-shell structured nanocomplexes for atherosclerosis management. Adv. Sci. (Weinh) 6 (12), 1900172. 10.1002/advs.201900172 31380165 PMC6662054

[B231] Wang Z. Wang Y. Jia X. Han Q. Qian Y. Li Q. (2019). MMP-2-Controlled transforming micelles for heterogeneic targeting and programmable cancer therapy. Theranostics 9 (6), 1728–1740. 10.7150/thno.30915 31037134 PMC6485184

[B232] Wang H. Ding T. Guan J. Liu X. Wang J. Jin P. (2020a). Interrogation of folic acid-functionalized nanomedicines: The regulatory roles of plasma proteins reexamined. ACS Nano 14 (11), 14779–14789. 10.1021/acsnano.0c02821 33084315

[B233] Wang H. Liu Y. He R. Xu D. Zang J. Weeranoppanant N. (2020b). Cell membrane biomimetic nanoparticles for inflammation and cancer targeting in drug delivery. Biomater. Sci. 8 (2), 552–568. 10.1039/c9bm01392j 31769765

[B234] Wang J. Chin D. Poon C. Mancino V. Pham J. Li H. (2021a). Oral delivery of metformin by chitosan nanoparticles for polycystic kidney disease. J. Control Release 329, 1198–1209. 10.1016/j.jconrel.2020.10.047 33127449 PMC7904655

[B235] Wang L. Yang J. Li S. Li Q. Liu S. Zheng W. (2021b). Oral administration of starting materials for *in vivo* synthesis of antibacterial gold nanoparticles for curing remote infections. Nano Lett. 21 (2), 1124–1131. 10.1021/acs.nanolett.0c04578 33459020

[B236] Wang X. Cheng R. Zhong Z. (2021). Facile fabrication of robust, hyaluronic acid-surfaced and disulfide-crosslinked PLGA nanoparticles for tumor-targeted and reduction-triggered release of docetaxel. Acta Biomater. 125, 280–289. 10.1016/j.actbio.2021.02.044 33677162

[B237] Wang Y. Pisapati A. V. Zhang X. F. Cheng X. (2021). Recent developments in nanomaterial-based shear-sensitive drug delivery systems. Adv. Healthc. Mater 10 (13), e2002196. 10.1002/adhm.202002196 34076369 PMC8273148

[B238] Wang L. Dou J. Jiang W. Wang Q. Liu Y. Liu H. (2022). Enhanced intracellular transcytosis of nanoparticles by degrading extracellular matrix for deep tissue radiotherapy of pancreatic adenocarcinoma. Nano Lett. 22 (17), 6877–6887. 10.1021/acs.nanolett.2c01005 36036792

[B239] Wei X. Ying M. Dehaini D. Su Y. Kroll A. V. Zhou J. (2018). Nanoparticle functionalization with platelet membrane enables multifactored biological targeting and detection of atherosclerosis. ACS Nano 12 (1), 109–116. 10.1021/acsnano.7b07720 29216423 PMC5859122

[B240] Wei Y. Luo L. Gui T. Yu F. Yan L. Yao L. (2021). Targeting cartilage EGFR pathway for osteoarthritis treatment. Sci. Transl. Med. 13 (576), eabb3946. 10.1126/scitranslmed.abb3946 33441426 PMC8027922

[B241] Weiss H. L. Selvaraj P. Okita K. Matsumoto Y. Voie A. Hoelscher T. (2013). Mechanical clot damage from cavitation during sonothrombolysis. J. Acoust. Soc. Am. 133 (5), 3159–3175. 10.1121/1.4795774 23654418

[B242] Wu G. Wei W. Zhang J. Nie W. Yuan L. Huang Y. (2020). A self-driven bioinspired nanovehicle by leukocyte membrane-hitchhiking for early detection and treatment of atherosclerosis. Biomaterials 250, 119963. 10.1016/j.biomaterials.2020.119963 32334199

[B243] Xi J. Talaat M. Si X. A. Han P. Dong H. Zheng S. (2020). Alveolar size effects on nanoparticle deposition in rhythmically expanding-contracting terminal alveolar models. Comput. Biol. Med. 121, 103791. 10.1016/j.compbiomed.2020.103791 32568674

[B244] Xie R. Ruan S. Liu J. Qin L. Yang C. Tong F. (2021). Furin-instructed aggregated gold nanoparticles for re-educating tumor associated macrophages and overcoming breast cancer chemoresistance. Biomaterials 275, 120891. 10.1016/j.biomaterials.2021.120891 34051669

[B245] Yang Q. Peng J. Chen C. Xiao Y. Tan L. Xie X. (2018). Targeting delivery of rapamycin with anti-collagen IV peptide conjugated Fe_3_O_4_@Nanogels system for vascular restenosis therapy. J. Biomed. Nanotechnol. 14 (7), 1208–1224. 10.1166/jbn.2018.2588 29944096

[B246] Yang C. Mi X. Su H. Yang J. Gu Y. Zhang L. (2019). GE11-PDA-Pt@USPIOs nano-formulation for relief of tumor hypoxia and MRI/PAI-guided tumor radio-chemotherapy. Biomater. Sci. 7 (5), 2076–2090. 10.1039/c8bm01492b 30860522

[B247] Yang A. Qiao B. Strohm E. M. Cao J. Wang Z. Yuan X. (2020a). Thrombin-responsive engineered nanoexcavator with full-thickness infiltration capability for pharmaceutical-free deep venous thrombosis theranostics. Biomater. Sci. 8 (16), 4545–4558. 10.1039/d0bm00917b 32671366

[B248] Yang L. Zang G. Li J. Li X. Li Y. Zhao Y. (2020b). Cell-derived biomimetic nanoparticles as a novel drug delivery system for atherosclerosis: Predecessors and perspectives. Regen. Biomater. 7 (4), 349–358. 10.1093/rb/rbaa019 32793380 PMC7414994

[B249] Yao J. Yang Z. Huang L. Yang C. Wang J. Cao Y. (2021). Low-Intensity focused ultrasound-responsive ferrite-encapsulated nanoparticles for atherosclerotic plaque neovascularization theranostics. Adv. Sci. (Weinh) 8 (19), e2100850. 10.1002/advs.202100850 34382370 PMC8498883

[B250] Yetisgin A. A. Cetinel S. Zuvin M. Kosar A. Kutlu O. (2020). Therapeutic nanoparticles and their targeted delivery applications. Molecules 25 (9), 2193. 10.3390/molecules25092193 32397080 PMC7248934

[B251] Yhee J. Y. Im J. Nho R. S. (2016). Advanced therapeutic strategies for chronic lung disease using nanoparticle-based drug delivery. J. Clin. Med. 5 (9), 82. 10.3390/jcm5090082 27657144 PMC5039485

[B252] Yin M. Loyer X. Boulanger C. M. (2015). Extracellular vesicles as new pharmacological targets to treat atherosclerosis. Eur. J. Pharmacol. 763, 90–103. 10.1016/j.ejphar.2015.06.047 26142082

[B253] Yokoi K. Kojic M. Milosevic M. Tanei T. Ferrari M. Ziemys A. (2014). Capillary-wall collagen as a biophysical marker of nanotherapeutic permeability into the tumor microenvironment. Cancer Res. 74 (16), 4239–4246. 10.1158/0008-5472.Can-13-3494 24853545 PMC4134692

[B254] You D. G. Yoon H. Y. Jeon S. Um W. Son S. Park J. H. (2017). Deep tissue penetration of nanoparticles using pulsed-high intensity focused ultrasound. Nano Converg. 4 (1), 30. 10.1186/s40580-017-0124-z 29170724 PMC5676802

[B255] Yu Y. Wang Z. Wang R. Jin J. Zhu Y. Z. (2021). Short-term oral administration of mesoporous silica nanoparticles potentially induced colon inflammation in rats through alteration of gut microbiota. Int. J. Nanomed. 16, 881–893. 10.2147/ijn.S295575 PMC7872941 33574668

[B256] Yu Y. Li S. Yao Y. Shen X. Li L. Huang Y. (2023). Increasing stiffness promotes pulmonary retention of ligand-directed dexamethasone-loaded nanoparticle for enhanced acute lung inflammation therapy. Bioact. Mater 20, 539–547. 10.1016/j.bioactmat.2022.06.016 35846844 PMC9253482

[B257] Zenych A. Fournier L. Chauvierre C. (2020). Nanomedicine progress in thrombolytic therapy. Biomaterials 258, 120297. 10.1016/j.biomaterials.2020.120297 32818824

[B258] Zhang Y. Gao W. Chen Y. Escajadillo T. Ungerleider J. Fang R. H. (2017). Self-assembled colloidal gel using cell membrane-coated nanosponges as building blocks. ACS Nano 11 (12), 11923–11930. 10.1021/acsnano.7b06968 29116753 PMC6336496

[B259] Zhang B. Kim H. Wu H. Gao Y. Jiang X. (2019). Sonothrombolysis with magnetic microbubbles under a rotational magnetic field. Ultrasonics 98, 62–71. 10.1016/j.ultras.2019.06.004 31202970 PMC6710138

[B260] Zhang D. Y. Dmello C. Chen L. Arrieta V. A. Gonzalez-Buendia E. Kane J. R. (2020). Ultrasound-mediated delivery of paclitaxel for glioma: A comparative study of distribution, toxicity, and efficacy of albumin-bound versus cremophor formulations. Clin. Cancer Res. 26 (2), 477–486. 10.1158/1078-0432.Ccr-19-2182 31831565 PMC7050644

[B261] Zhang S. Xu W. Gao P. Chen W. Zhou Q. (2020). Construction of dual nanomedicines for the imaging and alleviation of atherosclerosis. Artif. Cells Nanomed. Biotechnol. 48 (1), 169–179. 10.1080/21691401.2019.1699823 31852323

[B262] Zhang W. Mehta A. Tong Z. Esser L. Voelcker N. H. (2021a). Development of polymeric nanoparticles for blood-brain barrier transfer-strategies and challenges. Adv. Sci. (Weinh) 8 (10), 2003937. 10.1002/advs.202003937 34026447 PMC8132167

[B263] Zhang X. Detering L. Sultan D. Luehmann H. Li L. Heo G. S. (2021b). CC chemokine receptor 2-targeting copper nanoparticles for positron emission tomography-guided delivery of gemcitabine for pancreatic ductal adenocarcinoma. ACS Nano 15 (1), 1186–1198. 10.1021/acsnano.0c08185 33406361 PMC7846978

[B264] Zhang D. Wang G. Yu X. Wei T. Farbiak L. Johnson L. T. (2022a). Enhancing CRISPR/Cas gene editing through modulating cellular mechanical properties for cancer therapy. Nat. Nanotechnol. 17 (7), 777–787. 10.1038/s41565-022-01122-3 35551240 PMC9931497

[B265] Zhang G. Wang Q. Tao W. Jiang W. Elinav E. Wang Y. (2022b). Glucosylated nanoparticles for the oral delivery of antibiotics to the proximal small intestine protect mice from gut dysbiosis. Nat. Biomed. Eng. 6 (7), 867–881. 10.1038/s41551-022-00903-4 35798834

[B266] Zhang X. Jin H. Huang X. Chaurasiya B. Dong D. Shanley T. P. (2022c). Robust genome editing in adult vascular endothelium by nanoparticle delivery of CRISPR-Cas9 plasmid DNA. Cell Rep. 38 (1), 110196. 10.1016/j.celrep.2021.110196 34986352 PMC8769807

[B267] Zhao Y. Xie R. Yodsanit N. Ye M. Wang Y. Gong S. (2020). Biomimetic fibrin-targeted and H(2)O(2)-responsive nanocarriers for thrombus therapy. Nano Today 35, 100986. 10.1016/j.nantod.2020.100986 33072177 PMC7561002

[B268] Zhao Z. Li M. Zheng L. Yang Y. Cui X. Xu T. (2022). Noninvasive transdermal delivery of mesoporous silica nanoparticles using deep eutectic solvent. J. Control Release 343, 43–56. 10.1016/j.jconrel.2022.01.019 35066098

[B269] Zhi D. Yang T. O'Hagan J. Zhang S. Donnelly R. F. (2020). Photothermal therapy. J. Control Release 325, 52–71. 10.1016/j.jconrel.2020.06.032 32619742

[B270] Zhong Q. Merkel O. M. Reineke J. J. da Rocha S. R. (2016). Effect of the route of administration and PEGylation of poly(amidoamine) dendrimers on their systemic and lung cellular biodistribution. Mol. Pharm. 13 (6), 1866–1878. 10.1021/acs.molpharmaceut.6b00036 27148629 PMC5469429

[B271] Zhong Y. Zhang Y. Xu J. Zhou J. Liu J. Ye M. (2019). Low-Intensity focused ultrasound-responsive phase-transitional nanoparticles for thrombolysis without vascular damage: A synergistic nonpharmaceutical strategy. ACS Nano 13 (3), 3387–3403. 10.1021/acsnano.8b09277 30855938

[B272] Zhou J. Li Y. S. Chien S. (2014). Shear stress-initiated signaling and its regulation of endothelial function. Arterioscler. Thromb. Vasc. Biol. 34 (10), 2191–2198. 10.1161/atvbaha.114.303422 24876354 PMC4169328

[B273] Zhou Q. Shao S. Wang J. Xu C. Xiang J. Piao Y. (2019). Enzyme-activatable polymer-drug conjugate augments tumour penetration and treatment efficacy. Nat. Nanotechnol. 14 (8), 799–809. 10.1038/s41565-019-0485-z 31263194

[B274] Zhou Y. Chen Z. Zhao D. Li D. He C. Chen X. (2021a). A pH-triggered self-unpacking capsule containing zwitterionic hydrogel-coated MOF nanoparticles for efficient oral exendin-4 delivery. Adv. Mater 33 (32), e2102044. 10.1002/adma.202102044 34216408

[B275] Zhou Z. Yeh C. F. Mellas M. Oh M. J. Zhu J. Li J. (2021b). Targeted polyelectrolyte complex micelles treat vascular complications *in vivo* . Proc. Natl. Acad. Sci. U. S. A. 118 (50), e2114842118. 10.1073/pnas.2114842118 34880134 PMC8685925

[B276] Zhu L. Liu H. W. Yang Y. Hu X. X. Li K. Xu S. (2019). Near-infrared fluorescent furin probe for revealing the role of furin in cellular carcinogenesis and specific cancer imaging. Anal. Chem. 91 (15), 9682–9689. 10.1021/acs.analchem.9b01220 31282656

[B277] Zhu T. Cui Y. Zhang M. Zhao D. Liu G. Ding J. (2020). Engineered three-dimensional scaffolds for enhanced bone regeneration in osteonecrosis. Bioact. Mater 5 (3), 584–601. 10.1016/j.bioactmat.2020.04.008 32405574 PMC7210379

[B278] Zhu Z. Zhai Y. Hao Y. Wang Q. Han F. Zheng W. (2022). Specific anti-glioma targeted-delivery strategy of engineered small extracellular vesicles dual-functionalised by Angiopep-2 and TAT peptides. J. Extracell. Vesicles 11 (8), e12255. 10.1002/jev2.12255 35932288 PMC9451528

[B279] Zinger A. Sushnitha M. Naoi T. Baudo G. De Rosa E. Chang J. (2021). Enhancing inflammation targeting using tunable leukocyte-based biomimetic nanoparticles. ACS Nano 15 (4), 6326–6339. 10.1021/acsnano.0c05792 33724785 PMC8155322

[B280] Zukerman H. Khoury M. Shammay Y. Sznitman J. Lotan N. Korin N. (2020). Targeting functionalized nanoparticles to activated endothelial cells under high wall shear stress. Bioeng. Transl. Med. 5 (2), e10151. 10.1002/btm2.10151 32440559 PMC7237145

